# Mustard oil-derived carbon dots for ultra trace Pb^2+^ detection and cell bioimaging applications

**DOI:** 10.1039/d6ra03057b

**Published:** 2026-09-29

**Authors:** Sonam Lhamu Sherpa, Sweta Gurung, R. Sai Prasad Goud, S. V. S. Nageswara Rao, Ritu Dixit, Parimal Das, Ravi Shankar Singh, Ajay Tripathi, Archana Tiwari

**Affiliations:** a Department of Physics, Institute of Science, Banaras Hindu University Varanasi-221005 India archana.tiwari@bhu.ac.in; b Department of Physics, Sikkim University Tadong Gangtok-737102 Sikkim India; c Centre for Advanced Studies in Electronics Science and Technology (CASEST), School of Physics, Centre for Nanotechnology (CFN), University of Hyderabad Hyderabad-500046 India; d Centre of Genetics Disorder, Institute of Science, Banaras Hindu University Varanasi-221005 India; e Department of Physics, Indian Institute of Science Education and Research Bhopal Bhopal-462066 India

## Abstract

Water-soluble blue fluorescent carbon soot-carbon dots (CS-CDs) were synthesized using mustard oil through a flame deposition method. This work investigates the effect of a confined combustion environment on particle formation. While particle size remained same even when the container volume was increased fourfold, a significant increase in defect density was observed. An intermediate volume of 1 L produces blue-shifted emission at 422 nm, compared to 430 and 428 nm for 0.5 L and 2 L, respectively, upon excitation at 350 nm. Microscopic and spectroscopic analyses revealed that as-synthesized CS-CDs possess a partially graphitized nanocrystalline core embedded within a highly disordered, defect-dense amorphous matrix. CS-CDs obtained from within the 2 L volume demonstrated sensitive and selective detection of Pb^2+^, achieving a detection limit as low as 1.57 nM in the linear range of 0–17 nM without surface functionalization despite their low quantum yield of 4%. Furthermore, the adsorption studies revealed multiple rate-limiting mass transfer processes, with an equilibrium adsorption capacity of 5.72 × 10^−4^ mg g^−1^ when added to 3.20 mg mL^−1^ CDs. MTT assays confirmed the non-cytotoxic nature of CS-CDs in C2C12 cell lines, validating their biocompatibility. These findings demonstrate a simple route to tune and tailor the characteristics of CS-CDs for designing eco-friendly, selective and biocompatible sensors for environmental monitoring.

## Abstract 2

Blue-emitting CS-CDs were derived from mustard oil soot, enabling sensitive Pb^2+^ detection *via* PL quenching, efficient pollutant adsorption, and cell imaging. It proves their multifunctional promise in sensing and biomedicine.

## Graphical abstract

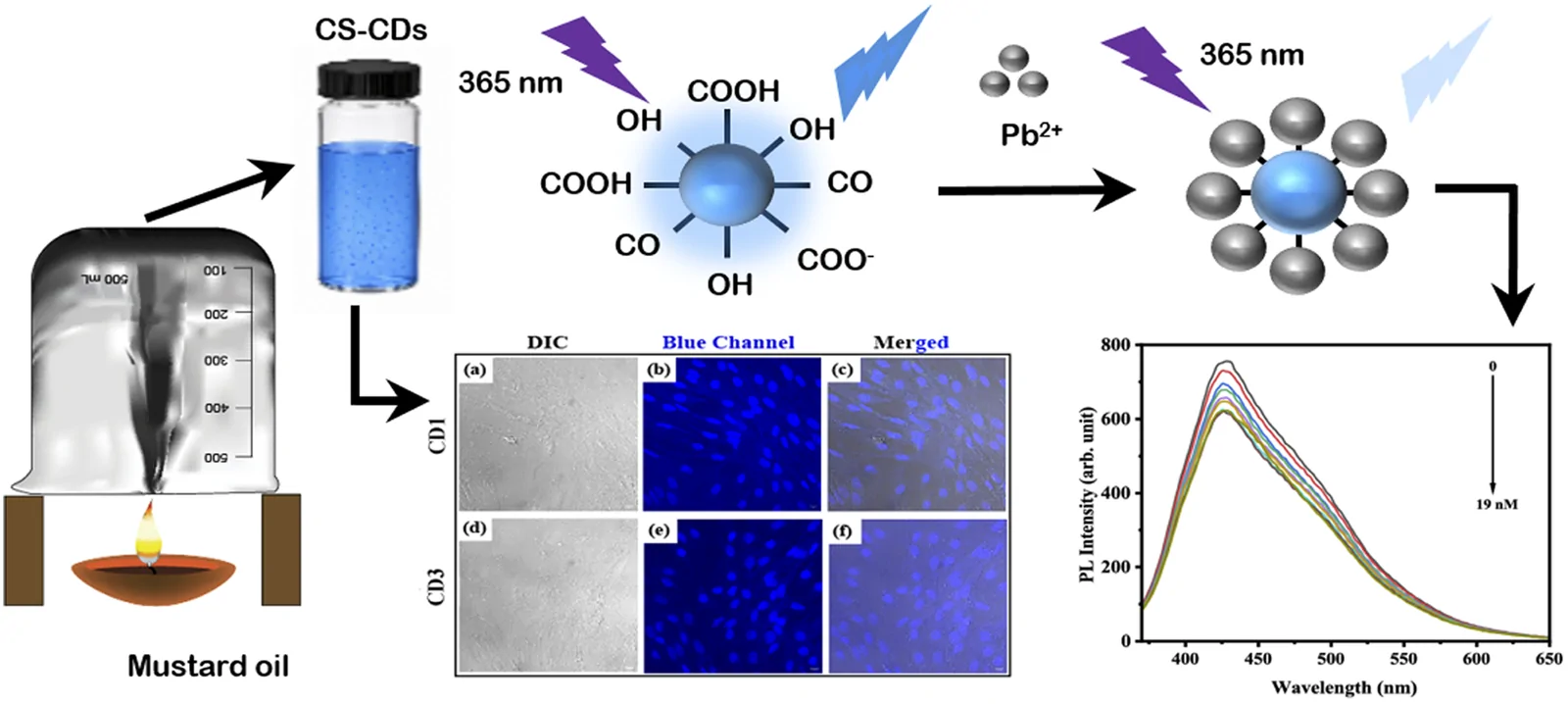

## 1 Introduction

Modern eye cosmetics, including many kajal or kohl products, often contain synthetic pigments, additives and inorganic compounds that may pose potential risks to ocular and systemic health.^1^ In particular, commercial eye products have been reported to contain high levels of lead and other inorganic substances raising health concerns.^2,3^ In contrast, traditional kajal prepared from naturally derived soot obtained through controlled combustion of high quality plant based oils, sometimes combined with herbal ingredients, has been used safely across cultures for centuries. This preparation avoids the use of toxic additives, relies on renewable and readily available ingredients and when prepared under hygienic conditions provides low-toxicity.^4^

In 2004, Xu and group accidentally discovered highly fluorescent nanoparticles during the purification of carbon nanotubes from carbon soot using gel electrophoresis.^5^ These nanoparticles were later coined as carbon quantum dots (CQDs) or C-dots (CDs) by Sun and coworkers in 2006.^6^ CDs offer emission tunability, water dispersibility, accessibility from abundant, cost-effective precursors, low toxicity and high photostability making them suitable for versatile applications including supercapacitors, light emitting diodes (LEDs), solar cells, drug delivery, bioimaging and notably, sensing.^7–11^ In recent years, researchers worldwide have increasingly focused on green and sustainable synthesis of CDs, utilizing carbon sources such as agricultural waste-biomass and plant materials.^11,12^ Beyond biomass-derived CQDs/CDs, carbon soot (CS), a carbonaceous by-product of incomplete combustion, abundant and rich in graphitic domains, is emerging as a valuable precursor due to its carbon abundance, fine particle size and minimal processing requirements. Notably, CDs have been derived from candle flames, kerosene lamps and various edible oils, emphasizing the potential of age old practices in modern nanomaterial synthesis.^13–15^ For instance, Chang *et al.*^16^ synthesized carbon nanoparticles from corn oil with a particle size 35–60 nm with high surface reactivity using a flame deposition process. Tyagi *et al.*^17^ reported the synthesis of activated mustard oil carbon soot *via* KOH-based chemical activation through a simple flame method, with a particle size of 50–80 nm.

Green biomass and soot-derived CDs have emerged as a highly promising analytical platform, offering exceptional selectivity, high sensitivity and a rapid response time for detecting environmental contaminants.^18^ The photoluminescence (PL) mechanisms of CDs are primarily governed by carbon core states, structural defects and surface traps associated with nitrogen- and oxygen-rich functional groups.^19^ This distinct photophysical property, combined with a large surface-to-volume ratio enables efficient light absorption and tunable PL emission facilitating strong interactions with chemical analytes and biological species. As a result, these CDs are frequently utilized for the development of highly selective optical sensors for the detection of heavy metal ions, environmental pollutants and small molecules.^18^ Lead (Pb^2+^), in particular, has a severe toxicity, non-biodegradable nature and ability to accumulate in living organisms often leading to hypertension, and illnesses in the immune system and the reproductive system.^20^ Despite this, many studies have reported a relatively high detection limit for Pb^2+^ using unmodified biomass CQDs/CDs, *e.g.* kans grass (*Saccharum spontaneum*) (376 nM),^21^ fenugreek seeds (9.34 µM)^22^ and sago industrial waste (7.49 µM).^23^ Some studies using Tulsi (*Ocimum sanctum*) leaves have reported lower nanomolar Pb^2+^ detection (0.59 nM).^11^ The hydrothermal synthesis of Tulsi derived-CQDs requires 4 h at 180 °C, excluding additional time for purification and post-synthesis processing. Achieving lower picomolar detection (0.19 nM) with watermelon juice-derived CQDs has typically required surface passivation or chemical functionalization.^24^ Also, Xing *et al.*^25^ reported Pb^2+^ detection of 1.55 nM, achieved with surface-modified walnut N-CQDs.

Inspired by the carbonaceous nature of traditional kajal, this work reports a flame deposition route for synthesizing blue emitting carbon soot carbon dots (CS-CDs) from mustard oil using exclusively deionized water, which takes only ∼4 h to complete the synthesis. This approach directly aligns with green chemistry principles where ambient atmospheric conditions are involved while utilizing natural biomass material and a safer solvent.^26^ This technique avoids toxic passivating reagents, high pressure vessels and energy-intensive thermal protocols like hydrothermal and solvothermal techniques.^10,27,28^ Beyond the eco-friendly precursor selection, this work systematically demonstrates the reaction volume controlled flame deposition (0.5 L, 1 L and 2 L) within the confined combustion environments, to modulate structural defect density and hence optical characteristics. The method is facile and efficient, producing CS-CDs with robust sensitivity towards the detection of toxic Pb^2+^ without any additional surface modification. A detailed study of the adsorption kinetics of Pb^2+^ on CS-CDs has also been performed. While some soot-derived carbonaceous nanomaterials show dose-dependent cytotoxicity,^28,29^ these CS-CDs exhibit excellent biocompatibility and cell-proliferation in C2C12 cells at concentrations even up to 1000 µg mL^−1^. This property enables their prolonged use in the biological system for imaging purposes, proving that these CS-CDs serve as robust, biocompatible fluorescent probes for live-cell diagnostic applications.

## 2 Experimental

### 2.1 Materials

Mustard oil, a clay lamp (diya) and cotton fiber were purchased from a commercial shop in Varanasi, India. Beakers of varying volumes (0.5 L, 1 L and 2 L) manufactured by Borosil were used. The chemical reagents used as the source for heavy metal ions include: calcium chloride (CaCl_2_, ≥98%), bismuth chloride (BiCl_2_, ≥98%), iron chloride (FeCl_3_, 99.99%), magnesium chloride (MgCl_2_, ≥ 98%), strontium chloride (SrCl_3_, ≥97%), lead chloride (PbCl_2_, ≥98%), cadmium chloride (CdCl_2_, 98%), arsenic trioxide (As_2_O_3_, ≥ 98%), aluminium chloride (AlCl_3_, ≥99%) and mercury chloride (HgCl_2_, ≥99.5%). All of them along with C2C12 murine myoblast cells were purchased from Merck, India. Dulbecco’s Modified Eagle’s Medium (DMEM), Fetal Bovine Serum (FBS) and phosphate-buffered saline (PBS) were obtained from Gibco, India. Dimethyl Sulfoxide (DMSO), Paraformaldehyde (PFA), Triton X-100, 4′,6-diamidino-2-phenylindole (DAPI) and 1,4-diazabicyclo[2.2.2]octane (DABCO) were purchased from Sigma-Aldrich, India. 3-(4,5-dimethylthiazol-2-yl)-2,5-diphenyl tetrazolium bromide (MTT) was procured from Himedia, India.

### 2.2 Synthesis of CS-CDs

To synthesize CS-CDs, the flame deposition method was adapted from Chang *et al.*^16^ where they have utilized the corn oil to collect carbon nanoparticles inside an enclosed 2 L beaker without post-synthesis purification. In our work, we collected the soot by burning mustard oil poured in a clay lamp using a soaked cotton wick, as shown in the schematic illustration Fig. 1. Initially, the clay lamp was filled with 5 mL of mustard oil and the hand twisted cotton wick (prepared from a standardized mass of 100 mg of commercial cotton with a fixed length of 12 cm) was dipped in to soak, following which the wick tip of 1.5 cm was ignited and allowed to burn statically through capillary action under ambient temperature. Upon stabilization of the flame after a minute of continuous oil absorption, the beaker was placed inverted on top of the clay lamp to collect the soot. To allow air flow, a small space of 3 cm was maintained in between the burning clay lamp and the beaker. After exhaustion of the oil, the beaker was removed and allowed to cool naturally. Thereafter, the deposited soot was dispersed in 200 mL of deionized water and sonicated vigorously in a hot water bath for 30 min, followed by 10 min of probe sonication. Once the soot was mixed uniformly, the solution was sequentially filtered through a fine Whatman filter paper of pore size 411 µm to extract large, insoluble carbonaceous aggregates, followed by micro syringe filtration through a 0.22 µm nylon membrane. The obtained nanoparticles were then labeled as CS-CDs. This standardized methodology was replicated across different batches by systematically altering the volume configuration of the collection beakers: 0.5 L, 1 L and 2 L, producing CS-CDs labeled as CD1, CD2 and CD3, respectively. The larger 2 L beaker provided greater head-space increasing the residence time of the smoke and resulting in higher soot deposition. In comparison, the smaller beakers allowed smoke to escape more rapidly, leading to lower soot collection. In order to confirm reproducibility, an identical synthesis protocol was adopted in two batches (*n* = 2). Furthermore, to ensure that the synthesized CDs are independent of the specific brand of oil used as a precursor, control experiments were conducted using two commercial oil brands: oil 1 (Fortune) and oil 2 (Parmatma). The total burning time required for the complete consumption of 5 mL of oil was recorded across all the batches and accounted to be 34 ± 1 min. The final mass per volume stock concentration of the aqueous solutions were determined using pycnometric density measurement and were found to be 2.96 ± 0.60 mg mL^−1^ (CD1), 1.01 ± 0.52 mg mL^−1^ (CD2) and 3.20 ± 0.30 mg mL^−1^ (CD3). These solutions were stored in an aqueous medium for further characterization.

**Fig. 1:**
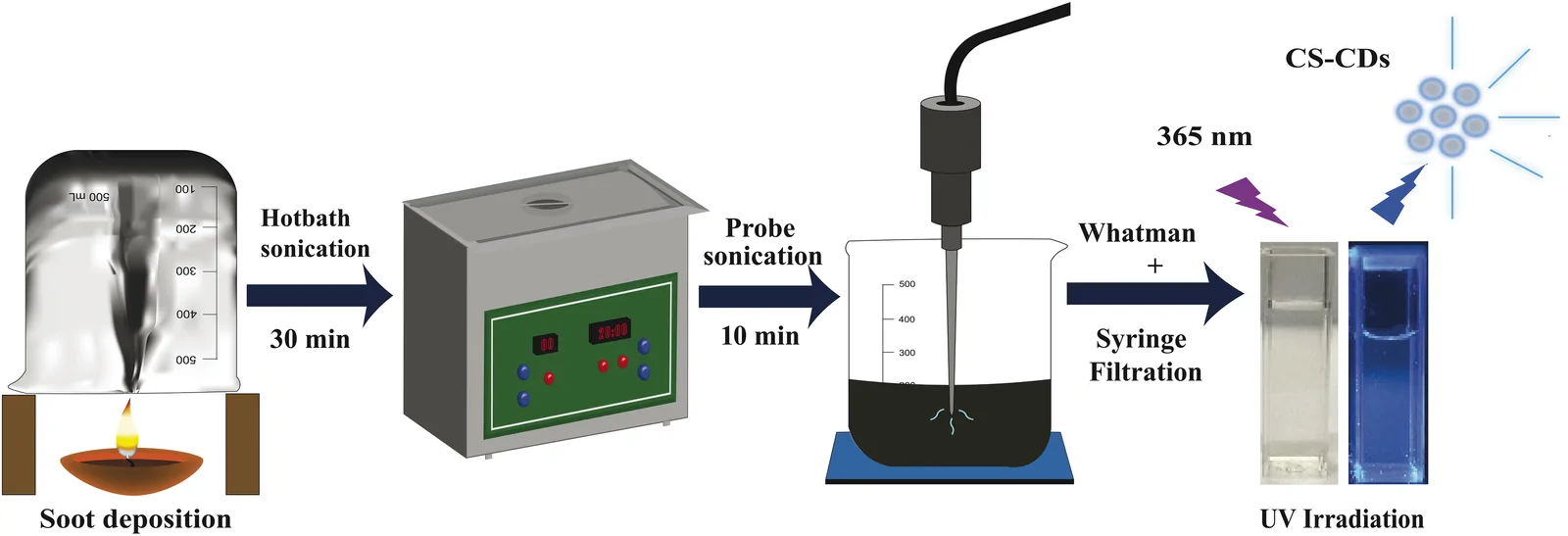
Schematic illustration of the synthesis of blue fluorescent CS-CDs.

### 2.3 Instrumentation and characterization

UV-Visible absorption characterization was carried out using a Lambda 25 PerkinElmer spectrophotometer in the wavelength range 200–900 nm. Photoluminescence (PL) and photoluminescence excitation (PLE) spectra were recorded in a PerkinElmer LS45 fluorescence spectrometer. The average size distribution of the particles was examined using a JEM-2100 HR transmission electron microscope (TEM) and Zeiss GeminiSEM 560. ImageJ and GATAN Microscopy Suite software were used to analyze the obtained micrographs. For elemental analysis, energy dispersive analysis (EDAX) was performed on a Carl Zeiss Microscopy GmbH. The x-ray diffraction (XRD) pattern was recorded using a PAnalytical XPert pro diffractometer with the aid of Cu Kα radiation (*λ* = 1.54 Å). For Raman spectroscopy, a Renishaw inVia spectrometer was employed using 785 nm laser excitation. To analyze the presence of functional groups, the as-synthesized CDs were drop-casted on a coverslip and FTIR spectra were scanned using a PerkinElmer Spectrum 100 in the range 800–4000 cm^−1^. X-ray photoelectron spectroscopy (XPS) was carried out using a PHI5000 Versa Probe III scanning XPS microprobe equipped with a monochromatized Al Kα X-ray source (1486.6 eV photons). Wide survey spectra were recorded with a pass energy of 280 eV and high resolution core-level spectra were recorded with a pass energy of 58 eV.

### 2.4 Metal ion sensing

To conduct the selectivity experiment, 0.5 mL of 1 nM stock solutions of various metal ions (Ca^2+^, Bi^2+^, Fe^3+^, Mg^2+^, Sr^3+^, Pb^2+^, Cd^2+^, As^3+^, Al^3+^, Hg^2+^) were prepared and individually mixed with 2.5 mL of CD3. In order to investigate the sensing response and competitive quenching at a higher analyte concentration, parallel experiments were also performed using 10 µM stock solutions of the same set of metal ions under identical conditions (Fig. S10). For sensitivity analysis, Pb^2+^ solutions with concentrations up to 19 nM were prepared and each 0.5 mL was mixed with 2.5 mL of CD3. Throughout the experiment, solutions were mixed for 3 min and excited at 350 nm. The resulting PL spectra were then recorded in duplicate (*n* = 2) with error bars representing mean ± standard deviation, in the emission range 380–650 nm.

### 2.5 Adsorption experiment

The adsorption behavior of Pb^2+^ on CD3 (3.2 mg mL^−1^) was investigated using an initial Pb^2+^ concentration of 7 nM. PL spectroscopy was used to determine the Pb^2+^ concentration during the adsorption study. A calibration curve was first constructed by recording the PL intensity of CD3 mixed with known concentrations of Pb^2+^ (0–19 nM) after a fixed 3 min mixing period under 350 nm excitation (Fig. S1). For adsorption studies, PL spectra of the solution (CD3 + 7 nM Pb^2+^) were recorded at regular time intervals of 5 min, with a 3 min mixing period included within each interval, until equilibrium was achieved. The maximum PL intensity was then correlated with the obtained calibration curve to determine the residual Pb^2+^ concentration and evaluate adsorption capacity. Although Pb^2+^ adsorption required ∼45–60 min to achieve equilibration, the sensing experiments were performed after a consistent 3 min interaction time. This ensured that all PL responses were obtained under identical conditions, allowing reliable evaluation and comparison of Pb^2+^ concentrations throughout the experiments.

### 2.6 Cell viability assay

#### 2.6.1 Cell culture procedure

C2C12 cells were used in the experiments for checking cell viability and cellular uptake of CD1 and CD3 particles. They are immortalized mouse myoblast cell lines, widely used to study skeletal muscle differentiation and cellular responses, capable of differentiating into myotubes under low serum conditions. The cells were maintained in DMEM containing 10% FBS under standard conditions of 37 °C with 5% CO_2_ and were maintained at sub-confluent densities (around 70%) through regular passaging to prevent spontaneous differentiation.

#### 2.6.2 Treatment with CD1 and CD3 and assessment of cell growth by phase contrast microscopy

C2C12 myoblasts were seeded at an initial density of 1 × 10^5^ cells/well in 6-well tissue culture plates. The cells were exposed to varying concentrations of CD1 and CD3 ranging from 32 µg mL^−1^ to 250 µg mL^−1^ in complete growth medium. Cell viability and morphological changes were observed over a time course of 12–48 h post treatment. Following the observation of sustained cell viability at lower concentrations through the 48 h time point, the experimental parameters were extended to examine the viability at higher CDs concentrations (250–1000 µg mL^−1^ each). In parallel, an untreated control group of C2C12 myoblasts was maintained under identical culture conditions throughout the experimental time course, serving as a baseline reference for comparative assessment of cell viability and morphology. Cellular morphology was documented using phase-contrast microscopy (Leica Microsystems) before CDs treatment and after 24 h of CDs treatment.

#### 2.6.3 MTT assay

C2C12 murine myoblast cells were utilized to evaluate the cytotoxicity of CD1 and CD3. The cells were cultured in 96-well plates at a seeding density of 1 × 10^4^ cells/well and exposed to increasing concentrations of CD1 and CD3 (25–1000 µg mL^−1^) for 24 h. Additionally, untreated cells cultured under identical conditions were utilized as the control. Post-treatment, cell viability was assessed using the MTT assay. Following the treatment period, the culture medium was replaced with MTT solution (0.5 mg mL^−1^) and the cells were incubated for 4 h at 37 °C. The resulting formazan crystals were then solubilized with 100 µL of DMSO followed by 10 min of gentle agitation in dark conditions. Also, a no-cell control containing CDs alone (without cells) was included and used as a blank to correct for any intrinsic absorbance contributed by the CDs themselves during the measurement. Absorbance of the solubilized formazan was quantified spectrophotometrically at 570 nm using a microplate reader. Cell viability was determined using optical density (OD) through the following equation:1
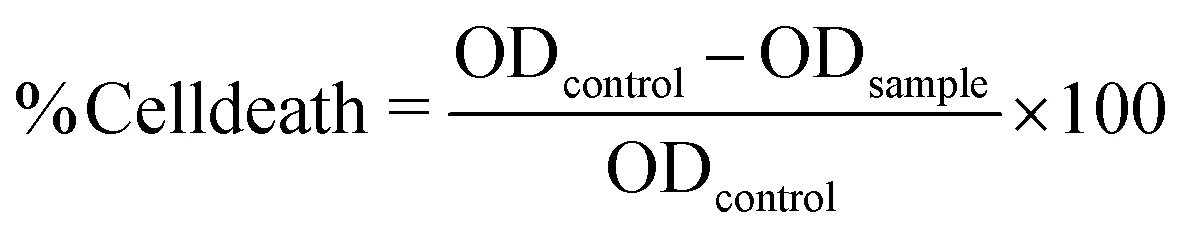


Data were acquired from two sets of independent experiments performed in triplicate (*n* = 3) and the pooled data were presented as mean absorbance with standard error.

#### 2.6.4 Confocal imaging of cells

Cells were cultured on methanol-treated coverslips placed in 6-well tissue culture plates. Following 12 h of cell adherence, the cultures were exposed to CD1 and CD3 at concentrations of 250 µg mL^−1^ and 500 µg mL^−1^ for another 24 h. Post-treatment, cells were subjected to sequential washing with PBS and fixed using 4% PFA at 4 °C for 15 min. After PBS washing, membrane permeabilization was performed using 0.1% Triton X-100 for 20–30 min under ambient temperature. Following dual PBS washes, nuclear counterstaining was conducted using DAPI at 1 µg mL^−1^ concentration for 15 min under dark conditions at room temperature. After subsequent PBS washing, specimens were mounted using DABCO anti-fade mounting medium and sealed with nail varnish. A confocal laser scanning microscope (ZEISS LSM 780) was employed to obtain high-resolution images. Additionally, acquired image and PL intensity analysis were performed by ImageJ software and panels were arranged using Adobe Illustrator.

## 3 Results and discussion

### 3.1 Morphological and structural study

The TEM micrograph of CD1, shown in Fig. 2(a), displays the formation of quasi-spherical particles with an average size of 4 ± 1 nm, as shown in Fig. 2(d). During the soot preparation of CD1, short-range crystalline ordering was also detected in the form of onion-like structures (Fig. S2(b)).^17^ Using fast Fourier transform (FFT) and inverse FFT (IFFT) techniques in the HRTEM micrograph (Fig. 2(g)), CD1 reveals *d*-spacing of 0.27 nm at three different sites (marked by yellow boxes), corresponding to the (100) plane as shown in the extended FFT images.^27^ The TEM micrograph of CD2 (Fig. 2(b)) shows a clustered distribution of spherical particles with an average size of 3 ± 1 nm, as shown in Fig. 2(e). The corresponding HRTEM micrograph (Fig. 2(h)) confirms the existence of the (100) plane with an interplanar spacing of 0.25 nm.^27^Fig. 2(c) displays the TEM micrograph of CD3 with sparsely distributed spherical particles having an average size of 4 ± 1 nm, as shown in Fig. 2(f). The HRTEM micrograph shown in Fig. 2(i) reveals lattice fringes with *d*-spacing of 0.35 nm, which corresponds to the (002) plane. The presence of (100) and (002) lattice planes in the CS-CDs confirms the existence of sp^2^ hybridized carbon domains.^14^ From the TEM micrographs, it is clear that there is no change in morphology and size, yet carbon soot collected from the four times larger reaction volume yields a comparatively defective graphitic structure, which is in good agreement with the Raman results discussed later in this section. In addition to these, some sparse distribution of bigger particles is also seen which is attributed to the aggregation of the smaller particles (Fig. S2). The selected area electron diffraction (SAED) pattern of CD1 and CD3 (Fig. S3(a) and (b)) exhibits diffused ring features confirming their amorphous nature with no significant long-range crystallinity. Additionally, FESEM micrographs (Fig. S4) exhibit a rough surface morphology with particle aggregation at the micron or sub-micron scales.

**Fig. 2:**
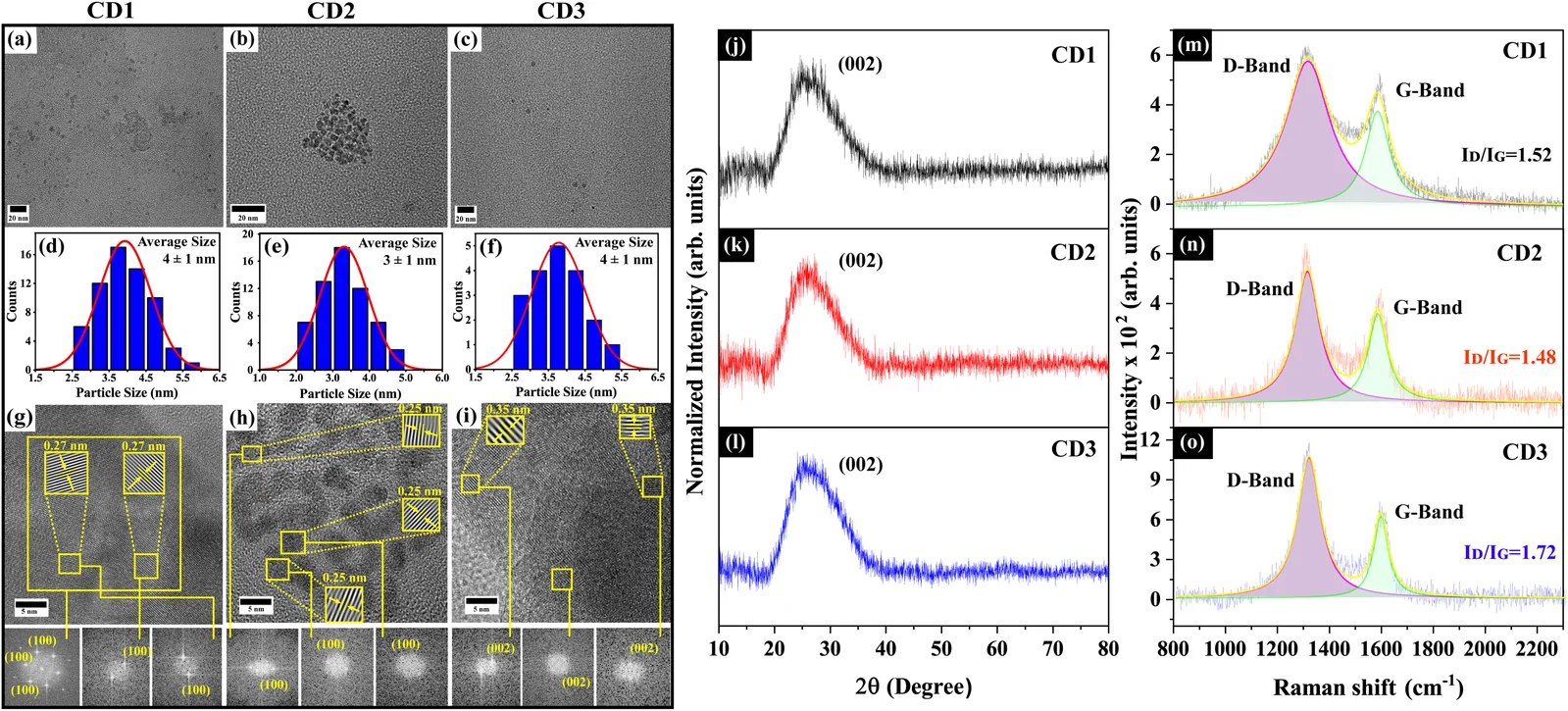
TEM micrographs of the CS-CDs: (a) CD1, (b) CD2, (c) CD3 and their corresponding particle size distribution in (d), (e) and (f); HR-TEM micrographs of the CS-CDs: (g) CD1, (h) CD2, (i) CD3, and their corresponding FFT and IFFT images (yellow box); XRD spectra of the CS-CDs: (j) CD1, (k) CD2 and (l) CD3 and Raman spectra of the CS-CDs: (m) CD1, (n) CD2 and (o) CD3.

The XRD patterns of CD1, CD2 and CD3 are shown in Fig. 2(j)–(l), respectively. All samples exhibit a broad diffraction peak centered at 25.5°, 25.8° and 25.9° for CD1, CD2 and CD3, respectively. These peaks are attributed to the (002) plane of the graphitic-like structure. The corresponding interplanar distances (*d*_002_), calculated using Bragg’s equation (2*d* sin θ = *nλ*), are found to be 0.35 nm for CD1 and 0.34 nm for CD2 and CD3. These values are larger than the reported graphitic spacing of 0.335 nm by Zhang *et al.*,^30^ which can be attributed to the presence of a short-range crystalline domain and defects in the CDs.^14^Fig. 2(m)–(o) display the Raman spectra of CD1, CD2 and CD3, respectively. Two prominent bands, D and G are observed with their peak positions summarized in Table 1. It is known that the D band is associated with disordered sp^3^ hybridized carbon atoms, indicative of structural defects, partial disorder or oxygen-containing functional groups, whereas the G band corresponds to the E_2g_ vibration mode of sp^2^ hybridized carbon atoms originating from the hexagonal graphitic core.^31^ The blueshift in the G band is attributed to the presence of the defects.^32^ As evident from the spectra, the D band exhibits a higher intensity (*I*_D_) than the intensity of the G band (*I*_G_) in all CS-CDs, reflecting a significant defect density. The intensity ratios *I*_D_/*I*_G_ of the Raman bands, listed in Table 1, are greater than unity for all samples. This indicates an increased concentration of surface defects or oxygen vacancies in the as-synthesized CS-CDs, which can play a crucial role as active sites in sensing applications.^33^

**Table 1:** Peak positions of D and G bands for the CS-CDs and their corresponding *I*_D_/*I*_G_ ratios

Sample	D-band (cm^−1^)	G-band (cm^−1^)	*I*_D_/*I*_G_
CD1	1315 ± 1	1585 ± 1	1.52
CD2	1317.4 ± 0.5	1586.2 ± 0.1	1.48
CD3	1312.1 ± 0.4	1588.1 ± 0.4	1.72

The FTIR spectra of CD1, CD2 and CD3 are shown in Fig. 3(a)–(c), respectively. All CS-CDs exhibit a broad absorption band in the range 3360–3746 cm^−1^ which is attributed to O–H stretching vibrations of hydroxyl groups and adsorbed water molecules. The absorption bands at 2920 cm^−1^ and 2851 cm^−1^ correspond to the asymmetric and symmetric stretchings of –CH_2_ bonds, respectively. The sharp peaks appearing at 1670 cm^−1^ and 1630 cm^−1^ are assigned to the stretching vibrations of carbonyl groups including C=C, C=O and –COOH bonds. The peaks located at 1470 cm^−1^ and 1222 cm^−1^ are attributed to C–H and C–O bonds, respectively. In addition, peaks at 1142 cm^−1^ and 1051 cm^−1^ indicate the presence of C–O–C/C–N stretches belonging to ester, ether and alcohol functional groups of the green precursor.^27,34^ These findings are consistent with the EDAX results (Fig. S5), confirming carbon (C), oxygen (O) and nitrogen (N) as the major constituent elements. Overall, the FTIR and EDAX data verify the functionalized, carbon-rich nature of the as-synthesized CS-CDs which enhances their water solubility and easy dispersibility.

**Fig. 3:**
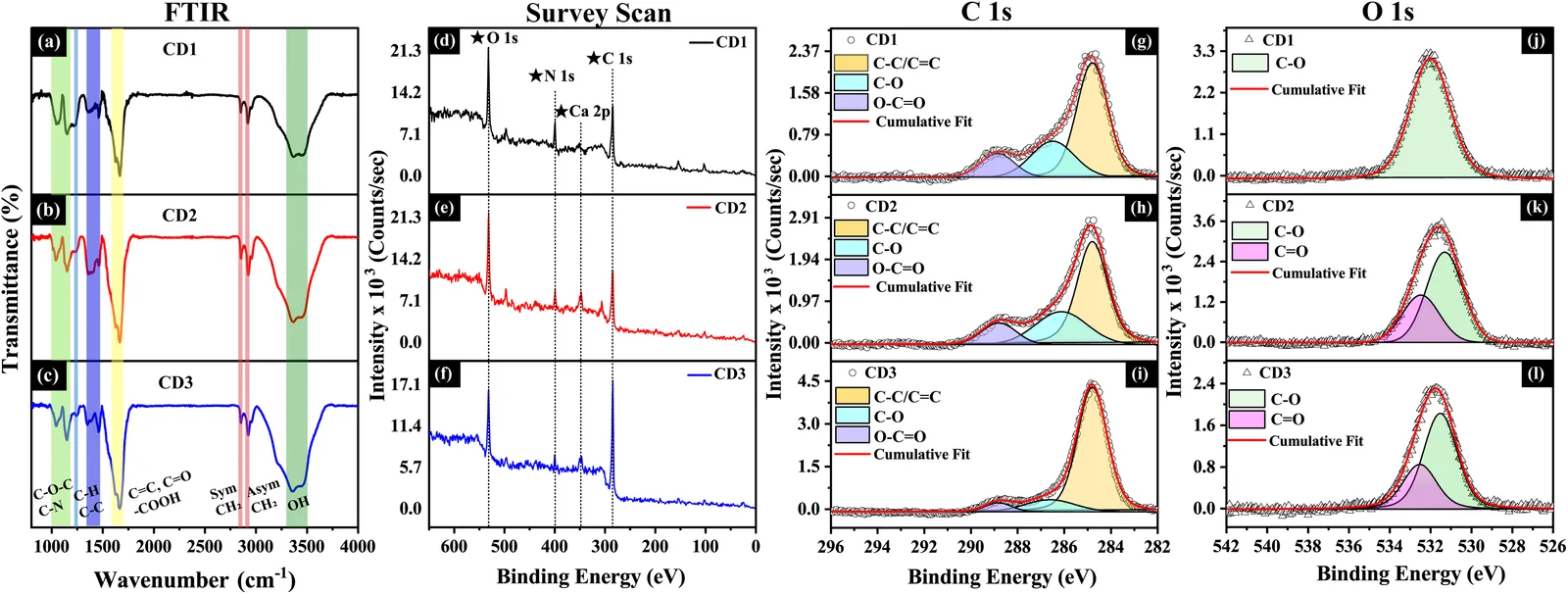
FTIR spectra of the CS-CDs: (a) CD1, (b) CD2, (c) CD3; XPS survey scan of the CS-CDs: (d) CD1, (e) CD2, (f) CD3; the narrow scan core level spectra of C 1s (g) CD1, (h) CD2 (i) CD3 and O 1s (j) CD1, (k) CD2 (l) CD3.

XPS was performed to understand the elemental composition and bonding of the CS-CDs. Fig. 3(d)–(f) present the wide survey scan of CD1, CD2 and CD3, respectively. All samples exhibit three prominent peaks at approximately 285 eV, 399.6 eV and 531.3 eV, corresponding to carbon (C 1s), nitrogen (N 1s) and oxygen (O 1s), respectively. In addition, a peak at 347.28 eV indicates the presence of trace calcium originating from the precursor. The elemental content from the survey spectra are determined to be 61.83% (CD1), 57.66% (CD2) and 70.65% (CD3) for C 1 s, 25.95% (CD1), 24.73% (CD2) and 15.69% (CD3) for O 1s, and 10.57% (CD1), 5.97% (CD2) and 5.07% (CD3) for N 1s. The high resolution C 1s spectra of CD1, CD2 and CD3 are shown in Fig. 3(g)–(i), respectively. For CD1, the C 1s spectrum is deconvoluted to three peaks at 284.8 eV, 286.5 eV and 288.8 eV, corresponding to C–C/C=C, C–O and O–C=O bonds, respectively. Similarly, for CD2, the spectrum exhibits three deconvoluted peaks at 284.8 eV, 286.12 eV and 288.8 eV, assigned to C–C/C=C, C–O and O–C=O bonds, respectively. The C 1s spectrum of CD3 also reveals three peaks at 284.8 eV, 286.6 eV and 288.8 eV, corresponding to C–C/C=C, C–O and O–C=O bonds, respectively.^35^ The high resolution O 1s spectra of CD1, CD2 and CD3 are presented in Fig. 3(j)–(l), respectively. The O 1s spectrum of CD1 shows a single peak at 532 eV, originating from the C–O group. In contrast, CD2 and CD3 exhibited asymmetry towards higher binding energies and were fitted with two peaks at 531.72 eV and 532.72 eV in CD2, and at 531.6 eV and 532.8 eV in CD3, which are assigned to C–O and C=O bonds, respectively.^35^ The relative peak areas (%) of the components of core-level spectra of C 1s and O 1s for the CS-CDs are tabulated in Table 2. The XPS results confirm the successful formation of CS-CDs, revealing a carbon rich framework with oxygen and nitrogen as a surface functionalities. In conclusion, as-synthesized CS-CDs exhibit a short ranged nanocrystalline core embedded within a highly disordered, defect-dense amorphous matrix.

**Table 2:** Relative peak area (%) obtained from XPS core-level spectra fitting of CS-CDs

Sample	C 1s (%)			O 1s (%)	
	C–C/C=C	C–O	O–C=O	C–O	C=O
CD1	67.70	27.30	5.00	100	–
CD2	60.16	27.74	12.10	78.02	21.98
CD3	83.50	11.80	4.70	81.30	18.67

### 3.2 Optical study

UV-Vis absorption spectra of the CS-CDs shown in Fig. 4(a) exhibit distinct absorption peaks at 278 nm, 270 nm and 280 nm for CD1, CD2 and CD3, respectively, with a miniature shoulder peak at 252 nm for CD1 and CD3. These peaks are attributed to a π–π* transition linked to sp^2^ hybridized aromatic carbon (C=C) bonds. Additionally, a broad shoulder peak centered at 323 nm is observed, corresponding to an n–π* transition of the carbonyl (C=O) groups.^8,36–38^ To confirm these observed electronic transitions, we have provided the first-derivative (d*A*/dλ) of all three CS-CDs in the supplementary information (Fig. S6). These spectra were further investigated to determine the corresponding optical band gap (*E*_g_) using Tauc and Davis–Mott relations: (*αhν*)^1/*n*^ = *A*(*E*_g_ − *hν*), where *α* is the optical absorption coefficient, *h* is the Planck constant, *ν* is the light frequency. *E*_g_ denotes the energy required to excite the electron, *A* is a constant and *n* is designated as the Tauc exponent. *n* represents the nature of transition that takes place in the material: *n* = 1/2 for direct (allowed) transition and *n* = 2 for indirect (allowed) transition. To calculate *E*_g_, we first inspected log(*αhν*) *vs.* photon energy (*hν*) to confirm direct allowed transition (*n* = 1/2), as shown in the inset of the Tauc plot Fig. 4(b)–(d) for CD1, CD2 and CD3, respectively.^39^ The Tauc plot reveals band gaps of 4.8 eV, 4.86 eV and 4.68 eV for CD1, CD2 and CD3, respectively. The lower energy shoulders observed in the Tauc plots, represent sub-band gaps and are attributed to the defect induced electronic states associated with surface functional groups, evident from multiple edges in absorption spectra.^27^ These defect states are observed at 4.31 eV and 3.64 eV in CD1, 3.90 eV and 3.56 eV in CD2 and 4.12 eV and 3.71 eV in CD3. Similar findings with *E*_g_ = 4.35 eV have been reported for diesel soot by Chaudhary *et al.*^40^ The presence and strength of the defect-related sub-band gap states correlate directly with Raman identified defects and complement the TEM findings.

**Fig. 4:**
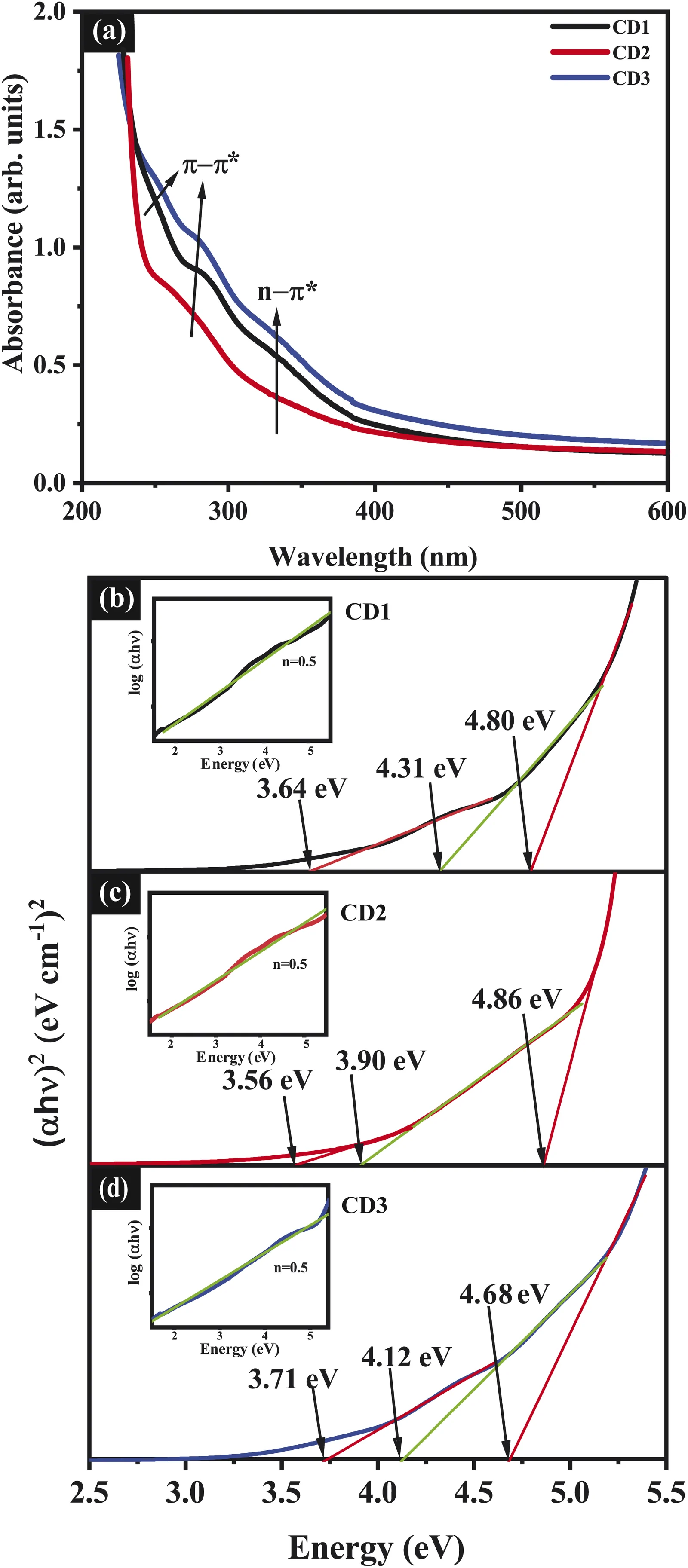
(a) UV-Vis absorption spectra of the CS-CDs: CD1 (Black), CD2 (Red) and CD3 (Blue); Tauc plots: (b) CD1, (c) CD2, (d) CD3 and their corresponding inset on the upper left showing log(*αhν*) *vs.* energy plots indicating direct transition with *n* = 1/2.

Fig. 5(a), (c) and (e) display the excitation dependent PL emission behavior of CD1, CD2 and CD3, respectively. The PL spectra were recorded over excitation wavelength ranges 290–350 nm for CD1, 290–365 nm for CD2 and 300–360 nm for CD3. Upon excitation at 350 nm, the maximum emission peaks are observed at 430 nm, 422 nm and 428 nm for CD1, CD2 and CD3, respectively. The observed volume-dependent shifts were found to be highly reproducible (*n* = 2) and independent of the precursor oil brand (Fig. S7 and S8). An additional emission peak at around 500 nm can be attributed to oxygen vacancy defects. As reported by Gan *et al.*,^41^ excitation dependent PL behavior arises from multiple emissive centers instead of originating from a single state, which is associated with the quantum confinement effect and surface defects in sp^2^ graphitic carbon domain. The excitation dependent normalized PL spectra shown in Fig. 5(b), (d) and (f) reveal energy shifts of 0.05 eV for CD1 and CD2 and 0.07 eV for CD3. These shifts further confirm the presence of multiple active emission sites in the CS-CDs.^41,42^

**Fig. 5:**
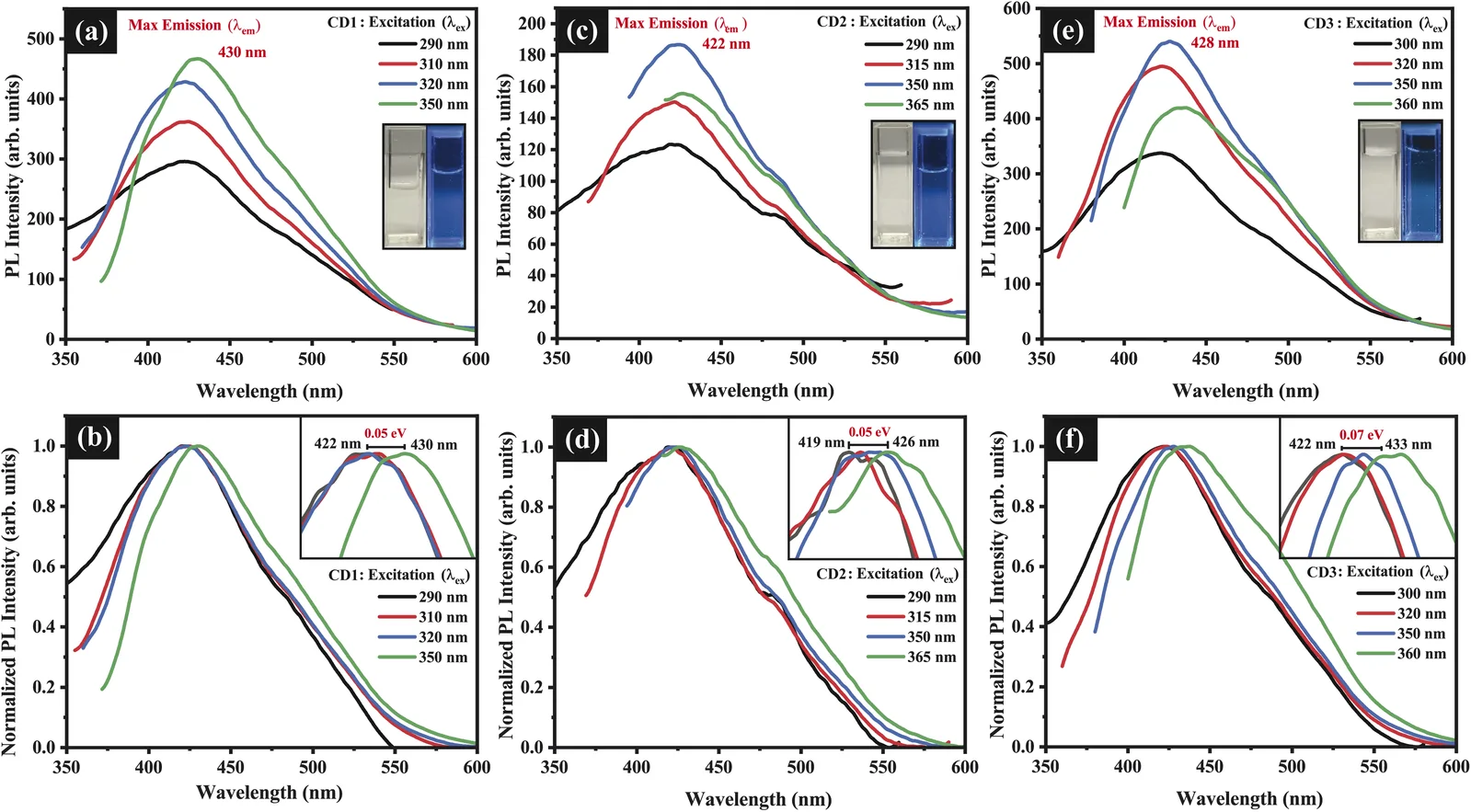
Excitation dependent PL emission spectra of (a) CD1 (290–350 nm), (c) CD2 (290–365 nm) and (e) CD3 (300–360 nm) with the insets showing the physical appearance of aqueous solutions of the CS-CDs under daylight (left) and 365 nm handheld UV-light (right); the corresponding normalized emission spectra of (b) CD1, (d) CD2 and (f) CD3 with the insets showing the shift in energy units.

Subsequently, PLE spectra were measured with emission monitored at 420 nm. The normalized PL spectra (dotted curves) and PLE spectra (smooth curves) are shown collectively in Fig. 6(a). The plots feature narrow Stokes shifts of approximately 0.73 eV, 0.72 eV and 0.71 eV for CD1 (black), CD2 (red) and CD3 (blue), respectively. Fig. 6(b) presents the corresponding energy level diagrams for CD1, CD2 and CD3, where the valence and conduction bands indicate the intrinsic optical band gaps of 4.86 eV, 4.80 eV and 4.68 eV, respectively. The observed PL emission from CD1, CD2 and CD3 occurs at energies lower than the optical bandgap, specifically at 2.88 eV (430 nm), 2.93 eV (422 nm) and 2.90 eV (428 nm) upon excitation at 3.54 eV (350 nm). This demonstrates that the electronic transition responsible for PL emission originates from defect states associated with surface functional groups and not directly from the band edge.^8^

**Fig. 6:**
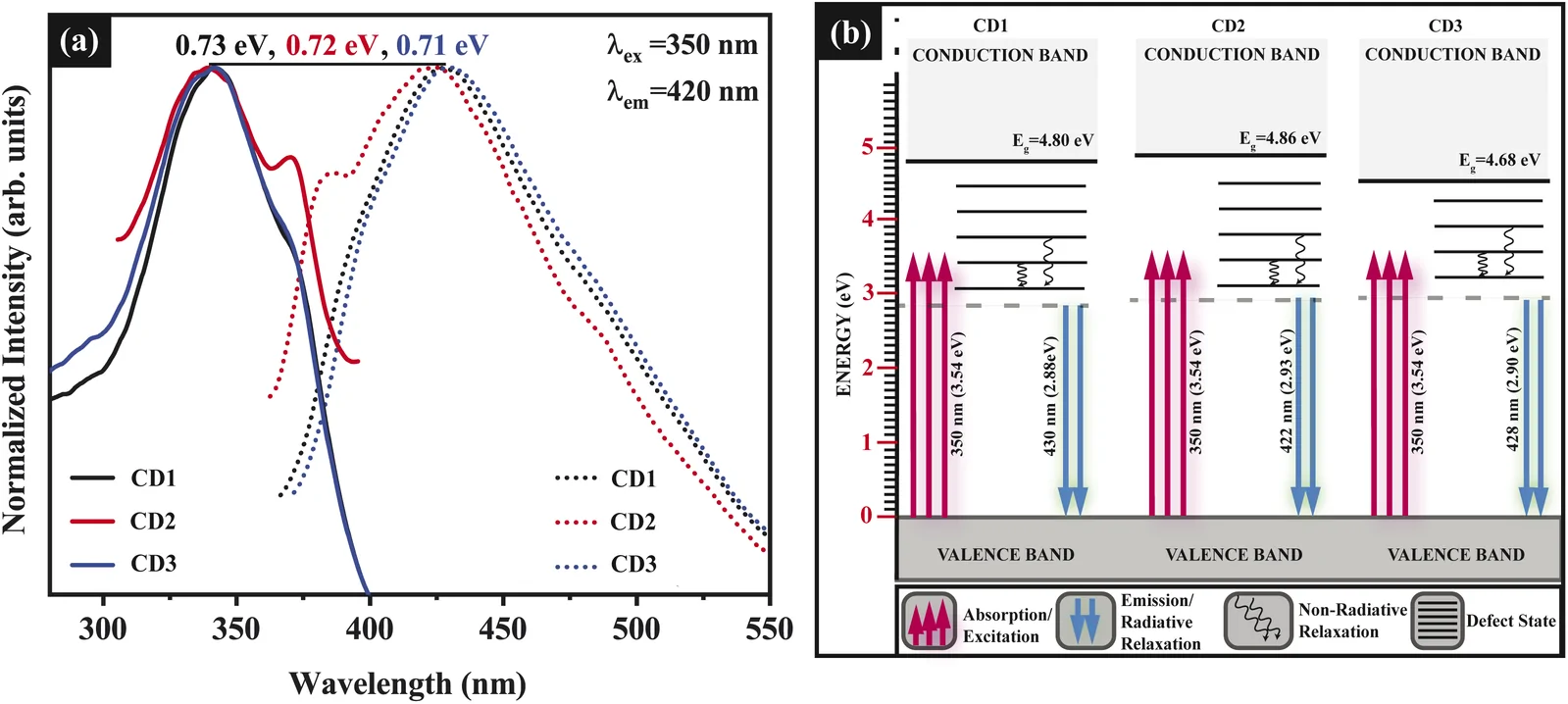
(a) PL emission spectra (*λ*_ex_ = 350 nm) and PL excitation spectra (*λ*_em_ = 420 nm) of the CS-CDs representing a red shift of 0.73 eV (CD1), 0.72 eV (CD2), 0.71 eV (CD3) and (b) energy level diagram representing the radiative and non-radiative transitions.

Additionally, we calculated the PL quantum yield (QY) of the CS-CDs considering quinine sulphate (QS) as the standard solution in 0.1 M sulphuric acid (H_2_SO_4_) with a known QY of 54% at 350 nm excitation. The PL spectral data were used to evaluate the QY of the as-synthesized CDs using following equation:2
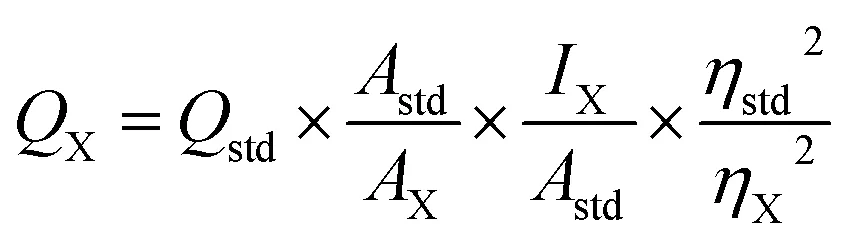
where, subscripts std and X denote standard solution (QS) and sample (CS-CDs), respectively. *A* denotes the absorbance, *I* indicates the integrated PL intensity and *η* refers to the solvent refractive index. Here, the ratio *η*_std_^2^/*η*_*X*_^2^ is equivalent to unity as both the solutions are in aqueous medium (*η*_std_ = *η*_*X*_ = 0.33).^43^ The QYs calculated with respect to QS for CD1, CD2 and CD3 are 4%, 2% and 4%, respectively.

## 4 Application in fluorescence spectroscopy

### 4.1 Selectivity and sensitivity analyses of Pb^2+^

Despite presenting equivalent PLQY (4%) to that of CD1, CD3 demonstrates a greater *I*_D_/*I*_G_ ratio (1.72), as evident from the Raman analysis. As a result, CD3 represents more concentrated structural defects and oxygen-containing functional groups which offers active binding sites for metal ion sensing. This is why CD3 has been utilized for further sensing applications. To evaluate selectivity, the PL response of blue-fluorescent CD3 toward various heavy metal ions (Ca^2+^, Bi^2+^, Fe^3+^, Mg^2+^, Sr^3+^, Pb^2+^, Cd^2+^, As^3+^, Al^3+^ and Hg^2+^) was systematically evaluated across 10 µM (Fig. S10) and 1 nM concentration regimes under 350 nm excitation (Fig. 7(a)). The results reveal a fluorescence quenching (turn-off) response for all tested metal ions, amongst which Pb^2+^ shows maximum change in PL intensity, indicating superior selectivity of CD3 towards Pb^2+^ across both concentration regimes. Notably, the relative quenching response and selectivity toward Pb^2+^ were far more pronounced and distinguishable at 1 nM than at 10 µM. This is why the 1 nM concentration of Pb^2+^ has been chosen for detailed sensitivity and adsorption studies on CD3.

**Fig. 7:**
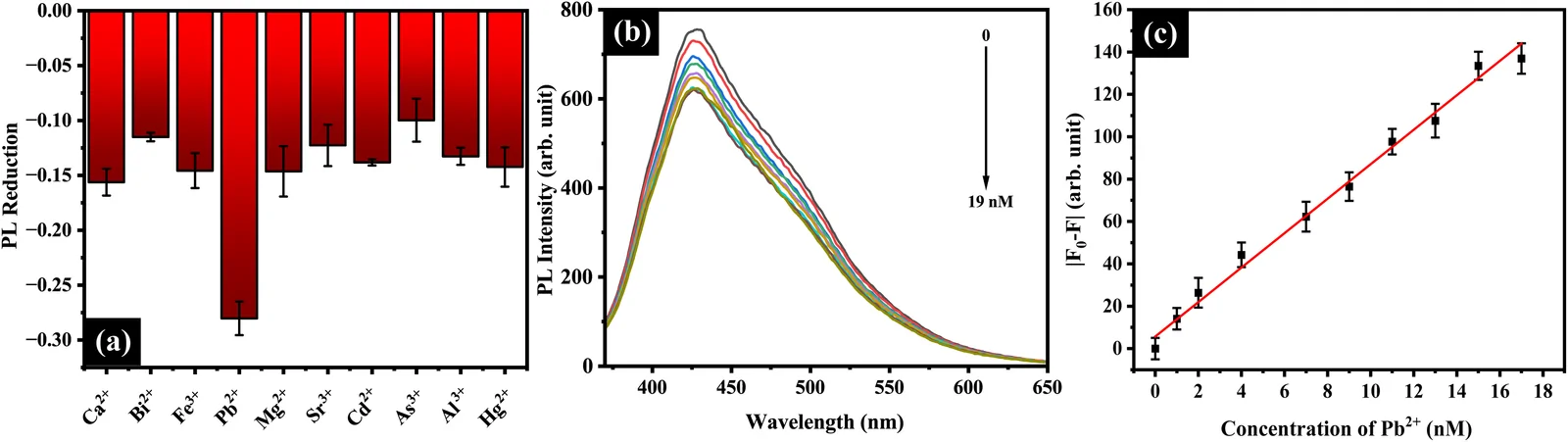
(a) Histogram showing relative PL intensity of CD3 when mixed with 1 nM concentration of different metal ions; (b) PL spectra recorded upon excitation of 350 nm when 0–19 nM concentration of Pb^2+^ is mixed with CD3 and (c) the linear relationship of quenching degree |*F*_0_ − *F*| against concentration of Pb^2+^.

Consequently, a sensitivity study was performed particularly for the detection of Pb^2+^ under optimized experimental conditions. The PL spectra were recorded at varying concentrations of Pb^2+^ (0–19 nM) under 350 nm excitation. As shown in Fig. 7(b), the gradual addition of Pb^2+^ to CD3 solution resulted in a steady decrease in the PL intensity. Furthermore, Fig. 7(c) demonstrates a good linear correlation between the relative PL intensity |*F*_0_ − *F*| and increasing Pb^2+^ concentration, where *F*_0_ and *F* signifies the PL intensity in the absence and presence of Pb^2+^, respectively. The corresponding Stern–Volmer plot within the linear range yields a high correlation coefficient (*R*^2^ = 0.988), confirming excellent analytical performance. The observed PL quenching behavior in the presence of metal ions can be attributed to concurrent mechanisms of static and dynamic quenching, charge transfer between the particles, formation of excimer and exciplex as the temporary excited state, *etc.*^44^ The selectivity and sensitivity of CDs depends on the precursor, synthesis conditions, surface functional groups and sensing mechanism. FTIR and XPS analyses confirm the presence of abundant oxygen-containing functional groups (–OH, –COO^−^ and –C–O–C) on the CDs surface. These groups act as active coordination sites for metal ions. In particular, Pb^2+^, being a heavy metal ion with vacant d-orbitals, exhibits a strong affinity toward these oxygen-donor ligands, forming stable non-fluorescent complexes. This interaction promotes photoinduced electron transfer from CD3 to Pb^2+^ and thus, results in effective PL quenching.^21,22,45^ The limit of detection (LOD) has also been assessed using the equation given below:3
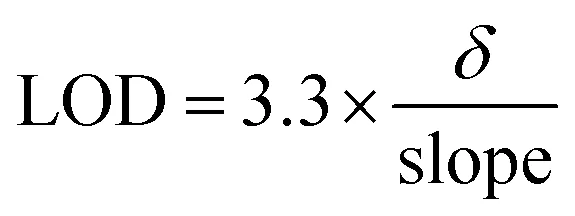
where *δ* is standard deviation and the value of slope is derived from the linear regression fit, as shown in Fig. 7(c).^46^ The as-synthesized CD3 exhibited an ultra low Pb^2+^ detection limit of 1.57 nM, enabling a high sensitivity to trace amounts of Pb^2+^. This detection limit is far lower than the reported blood Pb^2+^ levels at which clinical actions are recommended under World Health Organization (WHO) guidelines.^47^ As observed from the comparative assessment in Table 3, our CS-CDs achieve a competitive detection limit of 1.57 nM without requiring toxic precursors or multistep modifications. The CS-CDs significantly outperform several unmodified biomass probes, such as those derived from kans grass (376 nM)^21^ and fenugreek seed (9.34 µM).^22^ This difference arises because other systems exhibit lower densities of active donor groups, which limits the availability of effective coordination sites for Pb^2+^. Probes such as fenugreek seed CDs exhibit minimal active surface functionalities (–OH and C=O stretching vibrations) as shown by FTIR study. Other biomass-derived probes (*e.g.*, Kans grass, *Delonix regia* leaves and pomegranate peel CDs) also exhibit higher LODs despite the presence of various functional groups.^21,48,49^ In contrast to previous studies, which fail to quantify structural defects or their role, the current work demonstrates that an exceptionally high *I*_D_/*I*_G_ ratio (>1) and a high density of localized sp^3^ defects are critical to Pb^2+^ detection sensitivity. These defects, combined with surface-rich functionalities (–OH, –COO^–^, –C–O–C), create high-affinity coordination sites that enable rapid ground-state complexation with Pb^2+^. Although chemically passivated systems like ethylenediamine-modified watermelon juice (0.19 nM)^24^ or hydrothermally cooked Tulsi leaves (0.59 nM)^11^ achieve slightly lower detection limits, they require intensive energy inputs (180 °C for 4 h) or secondary synthetic agents. In contrast, our ambient flame deposition protocol operates without the use of sophisticated instrumentation and external reagents. Thus, it successfully bridges the gap between synthetic simplicity and ultra-sensitive nanomolar Pb^2+^ detection. This work confirms that a high PLQY is not a prerequisite for achieving a low detection limit. The sensing performance of CDs is governed not only by their PL intensity but also by the nature of their surface functional groups, the binding affinity towards the target ion (Pb^2+^) and the PL quenching mechanism.

**Table 3:** Comparison of green biomass-derived CDs for Pb^2+^ detection using various precursors

Materials/precursors	Sensor type	PL response	Linear range	LOD	References
Tulsi leaves CDs	Fluorometric	Turn-off	0.01–1 µM	0.59 nM	11
Kans grass CDs	Fluorometric	Turn-off	1–10 µM	376 nM	21
Fenugreek seed CDs	Fluorometric	Turn-off	—	9.345 µM	22
Sago industrial waste CDs	Fluorometric	Turn-off	—	7.49 µM	23
Ethylenediamine passivated watermelon juice CQDs	Fluorometric	Turn-off	0.01–50 µM	0.19 nM	24
Walnut green skin AGADES N-CQDs	Fluorometric	Turn-off	10 nM–1 mM	1.55 nM	25
*Delonix regia* leaves GCDs	Fluorometric	Turn-off	10–180 µM	3.3 nM	48
Agricultural waste CDs	Fluorometric	Turn-off	0.01–50 µM	16.93 nM	50
Pomegranate peel juice CQDs	Fluorometric	Turn-on	5–100 µM	18 µM	49
Guava leaves PG-CQDs	Fluorometric	Turn-off	0–50 µM	5.5 nM	51
Mustard oil CS-CQDs	Fluorometric	Turn-off	0–17 nM	1.57 nM	This Work

### 4.2 Adsorption studies

The adsorption study of Pb^2+^ was carried out on CD3. The adsorption capacity of Pb^2+^, *Q* (mg g^−1^), can be evaluated by the following equation:4
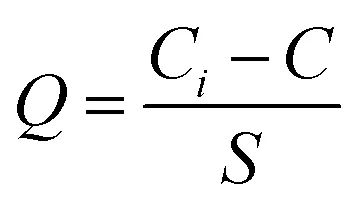
where, *C*_*i*_ and *C* (mg L^−1^) refer to the concentration of Pb^2+^ at initial and a specific time (min) after adsorption, respectively. *S* (g L^−1^) is the dose concentration of CD3 (3.20 mg mL^−1^).^46^Fig. 8(a) indicates the adsorption of Pb^2+^ on CD3, *Q* (mg g^−1^), as a function of contact time (min). It shows that the adsorption capacity rapidly increased during the initial 0–45 min and attains saturation thereafter. The quick uptake of Pb^2+^ by CD3 can be attributed to the high availability of the active sites and the rapid migration of ions to its proximity. The adsorption capacity rises with increasing Pb^2+^ concentration until a plateau like region is attained after 45 min, which can be ascribed to the depletion of active sites.^52^ The adsorption capacity of CD3 was found to be 5.72 × 10^−4^ mg g^−1^.

**Fig. 8:**
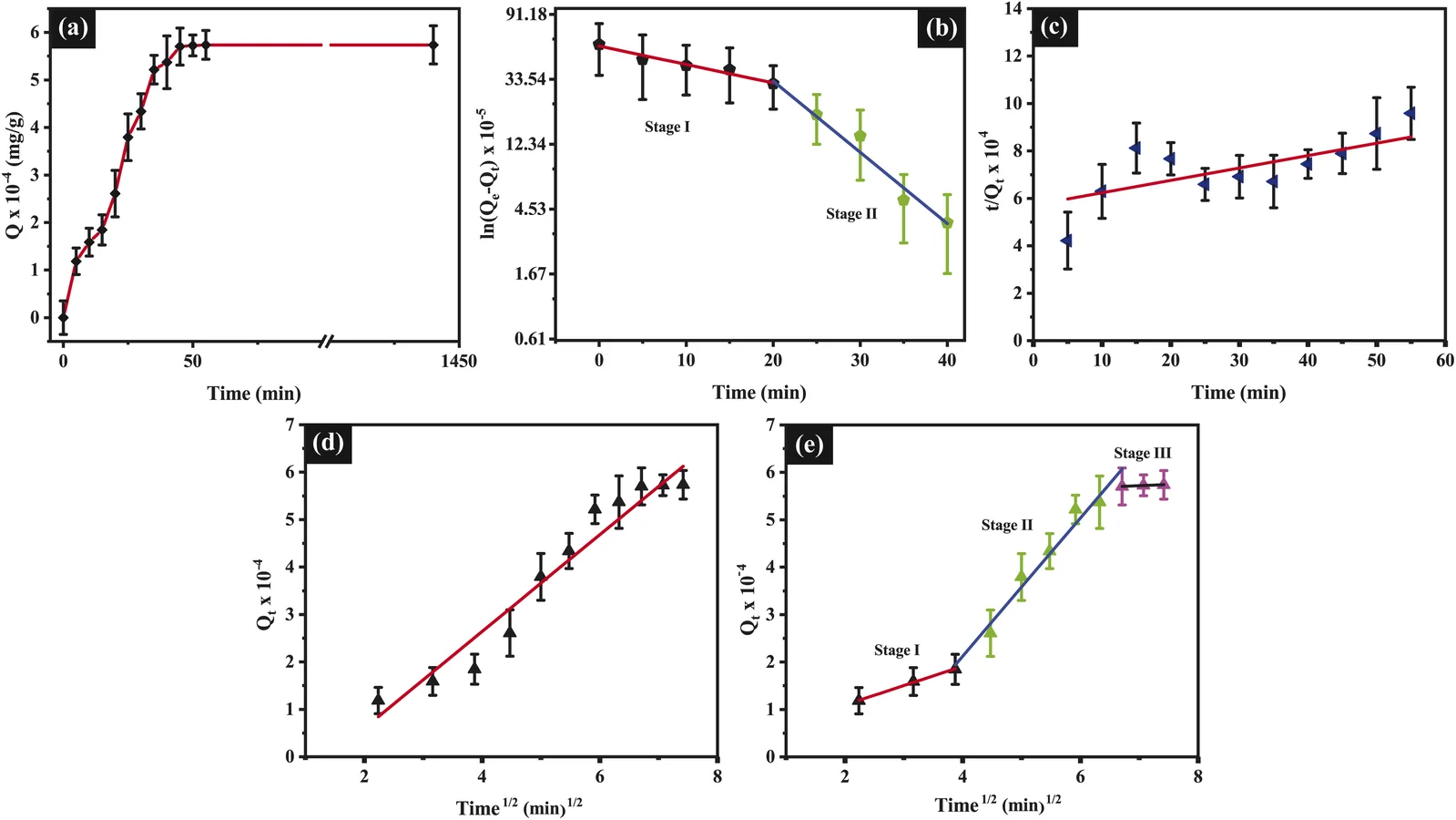
(a) Effect of contact time on the adsorption of Pb^2+^ onto CD3; kinetic models for the adsorption of Pb^2+^ on CD3: (b) pseudo-first order, (c) pseudo-second order (d) intraparticle diffusion with the straight line not intersecting origin of the coordinate system and (e) intra-particle diffusion explained with three linear stages.

For a comprehensive understanding of the adsorption kinetics, we investigated three kinetics models to predict the nature of the adsorption of Pb^2+^ on CD3. The models employed include: (i) a pseudo-first order (PFO) model, (ii) a pseudo-second order (PSO) model and (iii) an intraparticle diffusion (IPD) model. The PFO kinetic model can be described by Lagergren’s kinetic equation:

5 ln(*Q*_e_ − *Q*_*t*_) = ln *Q*_e_ − *K*_1_*t*

The PSO kinetic model or Ho and McKay kinetic model is given by the equation:6
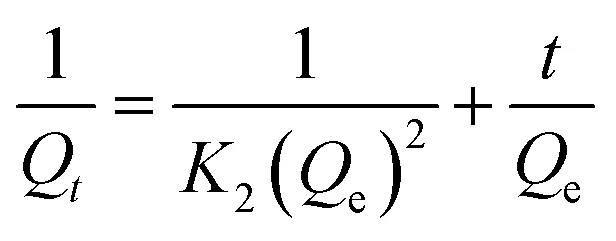


The IPD model can be depicted by the equation given below:

7 *Q*_*t*_ = *K*_3_*t*^0.5^ + [*C*]

where *Q*_*t*_ and *Q*_e_ (mg g^−1^) are the adsorption capacity at time *t* (min) and equilibrium adsorption capacity, respectively. *K*_1_ (min^−1^), *K*_2_ (g mg^−1^ min^−1^) and *K*_3_ (mg g^−1^ min^−0.5^) are the corresponding rate constants of PFO, PSO and IPD models, respectively. [*C*] is related to a boundary layer effect.^53^

The linearized form of various kinetic models employed to assess the adsorption of Pb^2+^ onto CD3 are presented in Fig. 8. The PFO model shown in Fig. 8(b) is fitted with two distinct linear regions. Unlike the conventional two step PFO behavior where a rapid initial stage is followed by a slower adsorption stage, our system demonstrates an inverse trend.^54^ The adsorption study in this work demonstrates a slower initial stage (stage I) within 0–25 min, characterized by a lower rate constant (*R*^2^ = 0.96) followed by faster adsorption (*R*^2^ = 0.94). As previously reported in piece-wise PFO kinetics, the initial phase corresponds to mass transfer and initial binding of analytes to low affinity sites, while the later phase is associated to the denaturation and reorganization of the analytes.^55^ These observations indicate that distinct mechanisms govern the adsorption process at different stages. The kinetic fitting using the PSO model shown in Fig. 8(c), exhibits a poor linear correlation (*R*^2^ = 0.38), suggesting that chemisorption is not the primary rate-limiting step in the adsorption process.

To further elucidate the rate-limiting mechanism, the IPD model was analyzed. According to Weber–Morris theory, the intraparticle diffusion governs the adsorption kinetics if and only if the linear plot derived from eqn (7) passes through the origin. As shown in Fig. 8(d), the linear fit does not intersect the origin, demonstrating that intraparticle diffusion is accompanied by boundary layer film effects. A multi-linear fit of the Q_*t*_*vs. t*^1/2^ plot (Fig. 8(e)), reveals three distinct diffusion kinetics. The first region (step I) exhibits a relatively slower adsorption with (*R*^2^ = 0.99), which is attributed to external mass transfer resistance, boundary layer diffusion and the delayed high affinity adsorption sites on CD3. The second region (stage II) represents a more pronounced increase in adsorption capacity (*R*^2^ = 0.95), corresponding to the diffusion of Pb^2+^ into the internal pores of the adsorbent, where intraparticle diffusion becomes more influential. The final region (stage III) is characterized by a plateau-like region (*R*^2^ = 0.89), which indicates the attainment of the equilibrium specifically due to complete site occupancy saturation and unavailability of further active sites.^53^ All kinetic parameters including adsorption capacity (*Q*_e_), kinetic rate constants (*K*_1_, *K*_2_, *K*_3_) of the different models, determination coefficients (*R*^2^) and boundary layer thickness constants [*C*] values are summarized in Table 4. In conclusion, Pb^2+^ uptake on natively functionalized CS-CDs is governed by the combined influence of film diffusion and surface interaction.^56^

**Table 4:** Kinetic parameters for adsorption of Pb^2+^ on CD3 (dose concentration: 3.20 mg mL^−1^) based on different kinetic models

Model	Stage	Parameter	Value
PFO	I	*K*_1_ (min^−1^)	0.028
		*Q*_e, exp_ (mg g^−1^)	0.00057
		*Q*_e_ (mg g^−1^)	0.00056
		*R* ^2^	0.96
	II	*K*_1_ (min^−1^)	0.109
		*Q*_e_ (mg g^−1^)	0.0029
		*R* ^2^	0.94
PSO		*K*_2_ (g mg^−1^ min^−1^)	4.8
		*Q*_e,exp_ (mg g^−1^)	0.00057
		*Q*_e_ (mg g^−1^)	0.0020
		*R* ^2^	0.38
IPD	I	*K*_3_ (mg g^−1^ min^−0.5^)	0.000041
		[*C*] (mg g^−1^)	0.000028
		*R* ^2^	0.99
	II	*K*_3_ (mg g^−1^ min^−0.5^)	0.00014
		[*C*] (mg g^−1^)	−0.00037
		*R* ^2^	0.95
	III	*K*_3_ (mg g^−1^ min^−0.5^)	0.0000048
		[*C*] (mg g^−1^)	0.00053
		*R* ^2^	0.89

### 4.3 *In vitro* cytotoxicity

To elucidate the biological effect of the as-synthesized CS-CDs, CD1 and CD3 were selected due to the comparable PLQY of 4%, which were approximately twice that of CD2 (∼2%). We examined the general morphology of cells grown in culture media and incubated with different concentrations (0–1000 µg mL^−1^) of CD1 and CD3 under a phase contrast microscopy. Phase contrast microscopy analysis revealed that C2C12 cells maintained viability and normal morphology at CD1 and CD3 concentrations up to 250 µg mL^−1^ (Fig. S11(a)), exhibiting no discernible cytotoxic effects compared to untreated controls. As shown in Fig. 9(A), an enhanced cellular proliferation was observed following 24 h of CD1 and CD3 exposure at concentrations ranging from 500–1000 µg mL^−1^, with proliferation rates exceeding those of untreated controls. This proliferative effect was particularly pronounced in CD3 treated cells, where more proliferation led to overconfluent cells, ultimately resulting in cellular detachment from the substrate due to space constraints. At 48 h post treatment of CDs, the overconfluent cells underwent contact inhibition and subsequently detached from the surface (Fig. S11(b)).

**Fig. 9:**
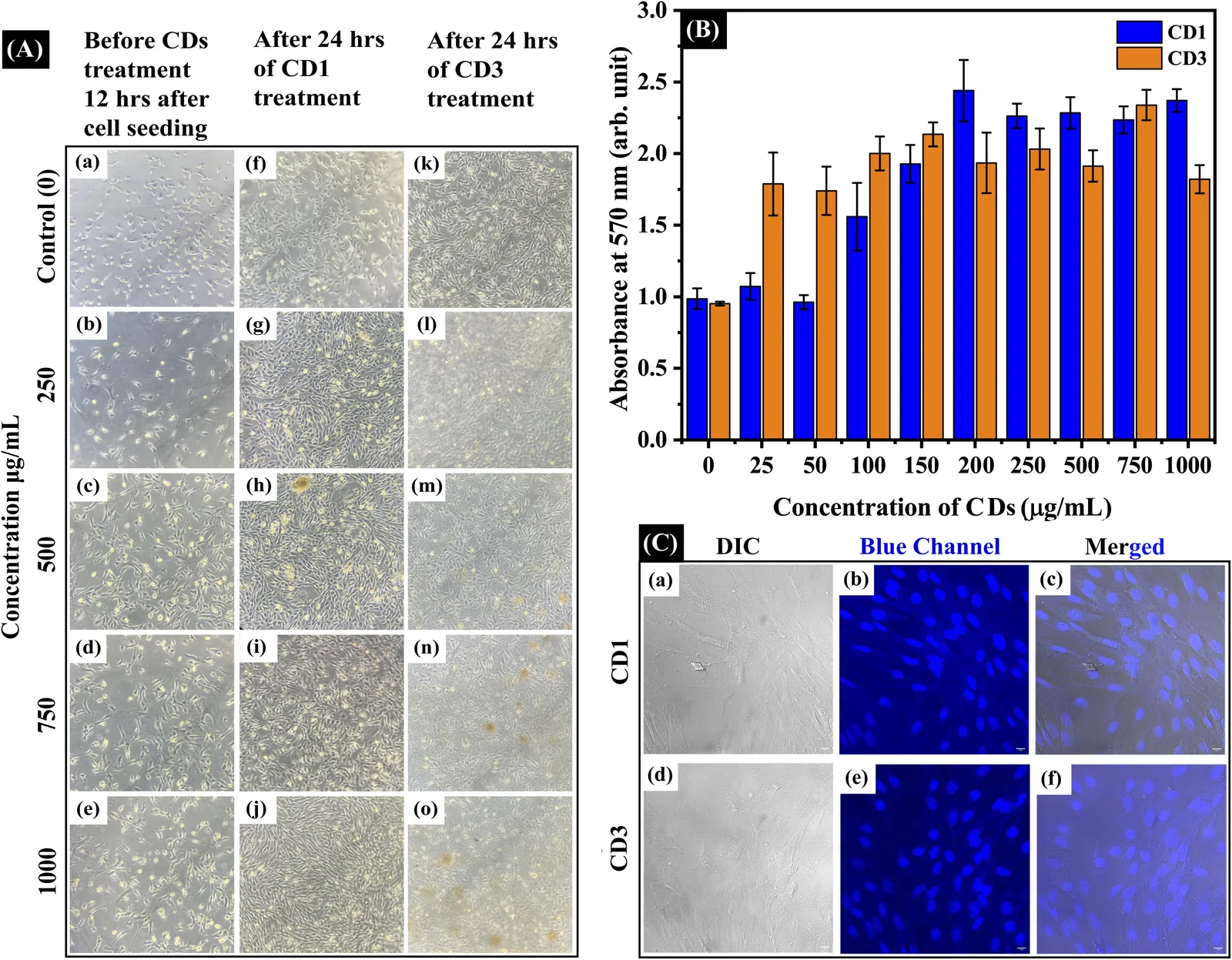
(A) Phase contrast microscopy of C2C12 cells after treatment with CD1 and CD3-250, 500, 750 and 1000 µg mL^−1^: (a–e) untreated cells 12 h after plating, (f and k) untreated cells 24 h after plating, (g–j) cells incubated with CD1 showing enhanced cell growth at all concentrations, (l–o) cells incubated with CD3 cells depicting relative overgrowth at 250 µg mL^−1^ and 500 µg mL^−1^ with overconfluency at higher concentrations. (B) Bar chart representing the dose dependent enhancement in C2C12 cells growth after 24 h of treatment with CD1 (blue) and CD3 (orange). The absorbance at 570 nm was plotted against the concentration of CS-CDs (µg mL^−1^) used. Data represented are an average of triplicates and error bar represents the standard error. (C) Confocal imaging of C2C12 cells treated with 250 µg mL^−1^ CS-CDs (a–c) CD1 and (d–f) CD3 after 24 h of incubation. DIC is the Differential Interference Contrast image of cells. The blue channel is the wavelength ranging from 405 nm to 491 nm which is within the range of the CS-CDs emission spectra and hence is able to detect the emission due to CS-CDs.

Previous studies on carbonaceous nanomaterials restricted cytotoxicity evaluations to narrow concentration ranges or observed strong anti-proliferative effects. For instance, Arkan *et al.*^29^ reported walnut-derived CQDs that suppressed cell proliferation at trace doses (1.25–5 µg mL^−1^). Similarly, Devi *et al.*^28^ evaluated amine-treated bike soot QDs (C1N) only up to 20 µg mL^−1^. Quantitative assessment of cellular viability following CD1 and CD3 exposure revealed distinctive proliferative effects. In cultures treated with CD1, no cytotoxicity was observed. Instead, a dose-dependent enhancement in cell viability was documented. The proliferation rate demonstrated a positive correlation with CD1 concentration within the range of 25–200 µg mL^−1^, after which the response became saturated as shown in Fig. 9(B) (blue). Parallel experiments with CD3 yielded comparable results presented in Fig. 9(B) (orange), exhibiting enhanced cell viability across the concentration range of 25–150 µg mL^−1^, ultimately reaching saturation in proliferation. No cytotoxic effect was observed across all tested concentrations for both CS-CDs variants.

### 4.4 Cellular imaging

Confocal imaging of CD1 and CD3 particles was performed post incubating cells with 250 µg mL^−1^ of both samples. Both CDs do not have any effect on the cell viability at this concentration as depicted in Fig. 9(B). So we have chosen a medium concentration of 250 µg mL^−1^ for the immunostaining at 24 h after CS-CDs treatment. The results indicate that both CS-CDs exhibited fluorescence after 24 h as shown in Fig. 9(C) and were nontoxic to the cells at all concentrations used in the MTT assay.

## 5 Conclusions

This study presents a facile and green approach to synthesize mustard-oil derived carbon dots (CS-CDs) revealing a link between common carbon sources and advanced nanomaterials. Despite a systematic investigation of the effect of reaction container volume on particle size, even a fourfold increase in container volume did not alter the particle size as evidenced by the TEM study. However, the container volume did influence particle aggregation which in turn affects the defect density in the material. The amorphous nature and defect rich framework confirmed *via* XRD and Raman studies, in combination with abundant surface functional groups identified through FTIR and XPS, and photoluminescent (PL) properties, demonstrates CS-CDs’ potential as an effective fluorescent probe. Amongst all CS-CDs, CD3 with significant defect density (*I*_D_/*I*_G_ = 1.72) and maximum QY (4%) was employed for selectivity and sensitivity studies with notable PL quenching towards Pb^2+^. It demonstrated ultra low Pb^2+^ detection of 1.57 nM in the linear range of 0–17 nM. Despite the low adsorption capacity of 5.72 × 10^−4^ mg g^−1^, the kinetic study displayed complex mechanisms instead of simple diffusion, involving multiple rate-limiting steps based on slower to faster adsorption and ultimately leading to the saturation of the available adsorption sites on the CS-CDs. Importantly, the MTT assay performed on C2C12 cell lines demonstrated a dose dependent increase in cell viability and negligible toxicity, suggesting that the CS-CDs are biocompatible. Therefore, the defect-engineered optical properties, high sensitivity and good biocompatibility makes CS-CDs a suitable candidate for applications in Pb^2+^ sensing and bioimaging.

While this study established a highly straightforward green and cost-effective pathway to synthesize naturally functionalized CDs, the sensitivity and selectivity of the soot-derived CDs for Pb^2+^ were validated in single-ion system. Future studies may be needed to check the sensing performance in a mixed ion environment to assess interference. Additionally, time-resolved photoluminescence (TRPL) decay kinetics can be pursued to comprehensively understand the lifetime dynamics of the defect state emission, paving the way for targeted applications in sustainable cosmetic formulations and environmental monitoring.

## Author contributions

Sonam Lhamu Sherpa: investigation, methodology, data curation, formal analysis, visualization, writing – original draft. Sweta Gurung: data curation, methodology, writing – review and editing. R. Sai Prasad Goud: Transmission electron microscopy. S. V. S. Nageswara Rao: writing – review and editing. Ritu Dixit: investigation, methodology, data curation, writing – original draft. Parimal Das: writing – review and editing. Ravi Shankar Singh: writing – review and editing. Ajay Tripathi: supervision, investigation, funding acquisition, project administration, writing – review and editing. Archana Tiwari: conceptualization, supervision, investigation, funding acquisition, project administration, Writing - review and editing.

## Conflicts of interest

There are no conflicts to declare.

## Supplementary Material

RA-OLF-D6RA03057B-s001

## Data Availability

All data supporting the findings of this study are available within the article and its supplementary information (SI). Additional datasets generated and analyzed during the current study are available from the corresponding author upon reasonable request. Supplementary information is available. See DOI: https://doi.org/10.1039/d6ra03057b.
