# Supplementary material for: Mustard oil-derived carbon dots for ultra trace Pb2+ detection and cell bioimaging applications
Source: RSC Adv. 2026 Sep 29;16(45):58525–38. doi: 10.1039/d6ra03057b (PMC13621487; doi:10.1039/d6ra03057b)
Supplement: Supplementary file 1 [file RA-016-D6RA03057B-s001.pdf]

Supplementary Information (SI) for RSC Advances.
This journal is © The Royal Society of Chemistry

Supplementary Information for Mustard oil-derived carbon dots for ultra trace Pb^{2+} detection and cell bioimaging applications †

Sonam Lhamu Sherpa[a], Sweta Gurung[b], R. Sai Prasad Gour[c], S. V. S. Nageswara Rao[c], Ritu Dixit[d], Parimal Das[d], Ravi Shankar Singh[e], Ajay Tripathi[b], Archana Tiwari[a,*]

[a] Department of Physics, Institute of Science, Banaras Hindu University, Varanasi 221005, India
[b] Department of Physics, Sikkim University, Tadong, Gangtok 737102, India
[c] Centre for Advanced Studies in Electronics Science and Technology (CASEST), School of Physics, Centre for Nanotechnology (CFN), University of Hyderabad, Hyderabad 500046, India
[d] Centre of Genetics Disorder, Institute of Science, Banaras Hindu University, Varanasi-221005, India
[e] Department of Physics, Indian Institute of Science Education and Research Bhopal, Bhopal-462066, India

* Corresponding author: archana.tiwari@bhu.ac.in

1 Calibration plot

Calibration plot is the standard curve plotted with signal response (PL intensity) against the known concentration of metal ions. This curve is linear fitted to determine the unknown concentration of Pb^{2+} quantitatively adsorbed by CD3. This curve as shown in Fig.S1 has R^2=0.98.

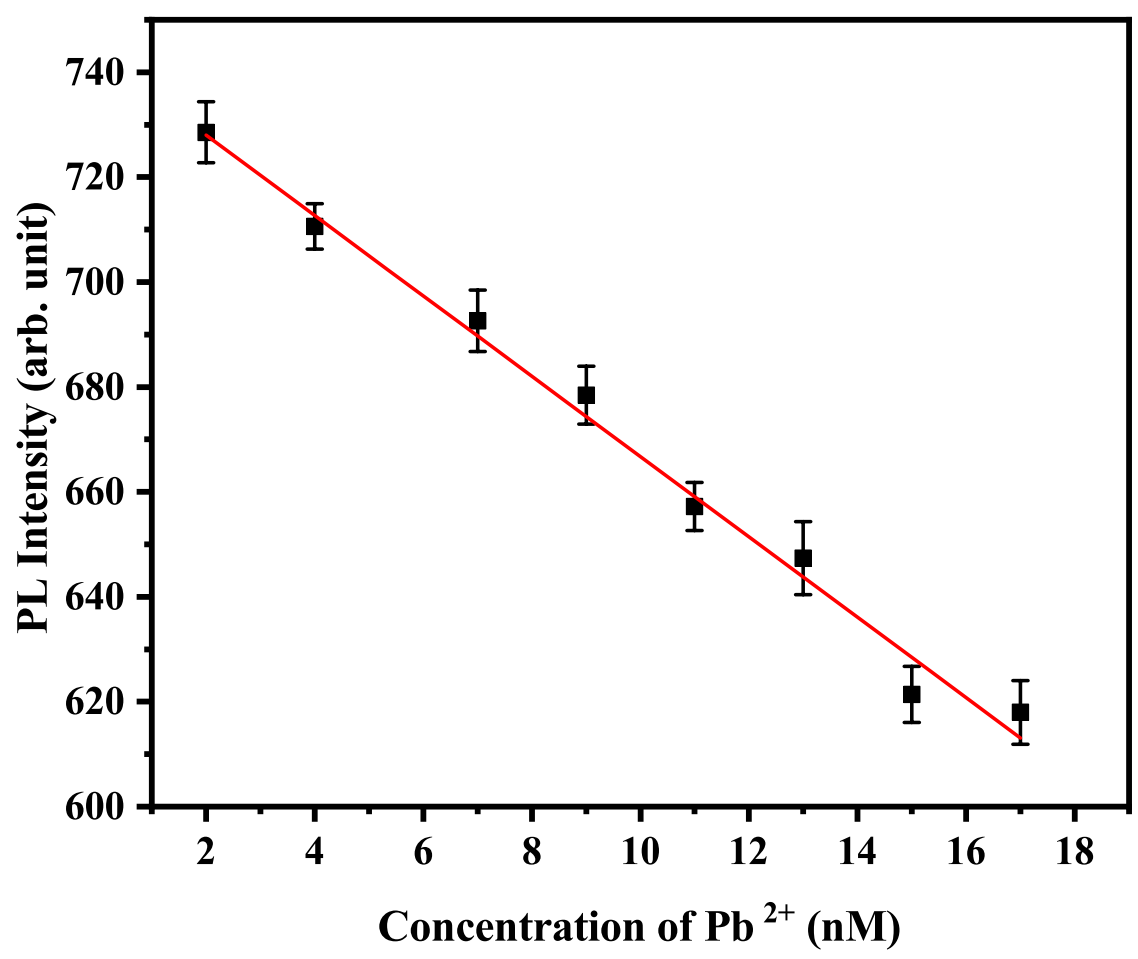

Figure S1 PL intensity versus concentration of Pb^{2+} for 3.20 mg/mL dose of CD3.

2 TEM, SAED and FESEM micrographs

Fig.S2(a), (d) and (e) reveals the fragile agglomerated particles in CD1, CD2 and CD3, respectively. It displays the irregular chain-like structures, branching to form an aggregates which clearly indicates the amorphous nature. Although CS-CDs exhibits an overall amorphous structure, but a trace of partial crystallinity is also evident in the form of interconnected onion-like structure, as shown in Fig.S2(b). Fig.S2(c) is the histogram showing average particle size of 69 ± 10 nm for CD1. Fig.S2(f), (g) and (h) are the supporting images used to calculate the average size of the particle for CD3 presented in Fig.2(c). The overall amorphous nature is further acknowledged by selected area diffraction pattern (SAED) shown in Fig.S3(a) and (b), corresponding to CD1 and CD3, respectively. It displays the diffused ring indicating poor crystallinity in CS-CDs. On the same note, FESEM micrographs in micron and sub-micron scales are shown in Fig.S4. It displays the spherical or flake like clusters, highlighting the agglomerated nature of CS-CDs.

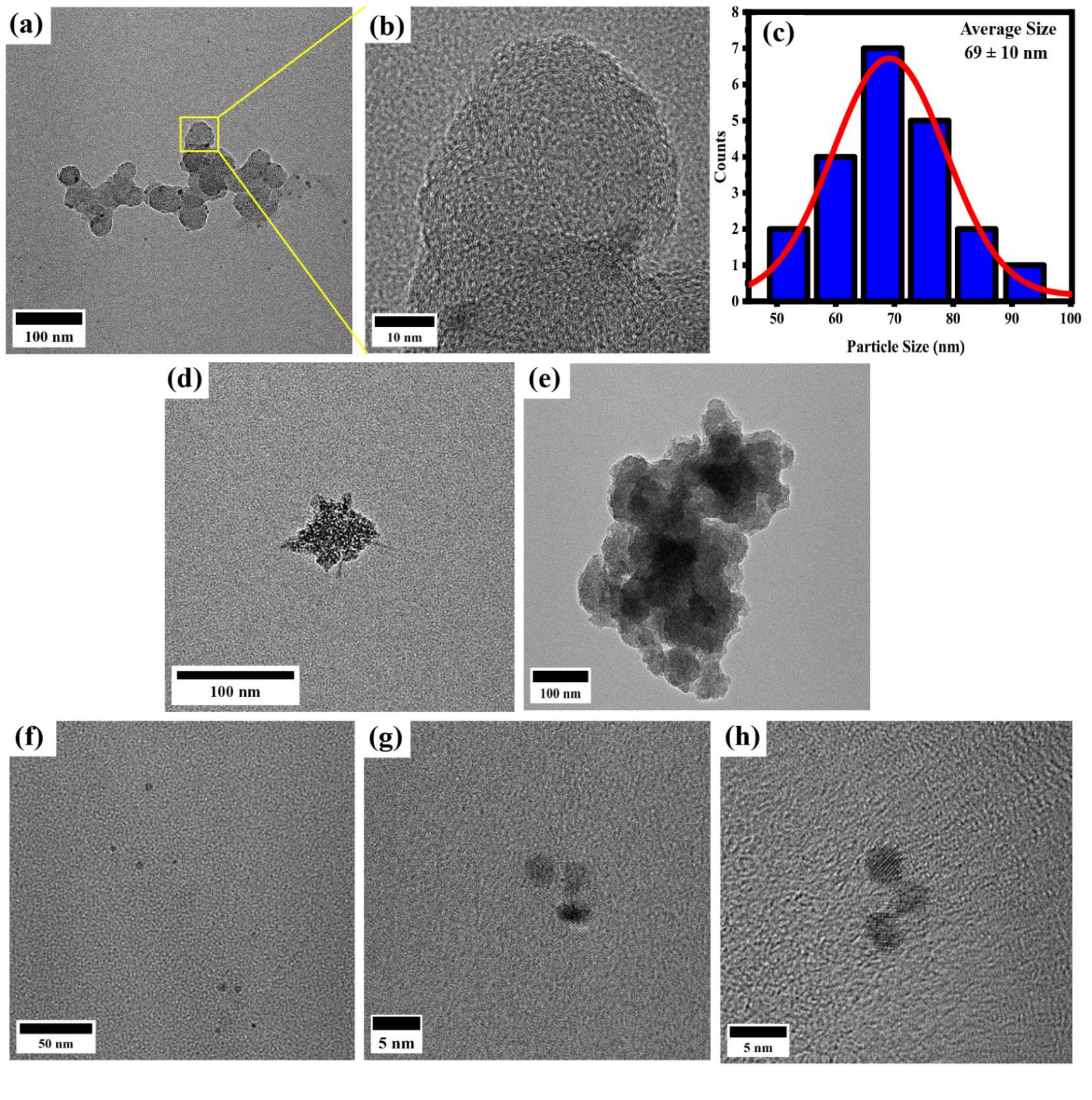

Figure S2 (a) TEM image of CD1, (b) onion-like nanoparticle formation in CD1, (c) particle size distribution of CD1 referring to (a), (d) TEM image of CD2 and (e,f,g,h) TEM images of CD3.

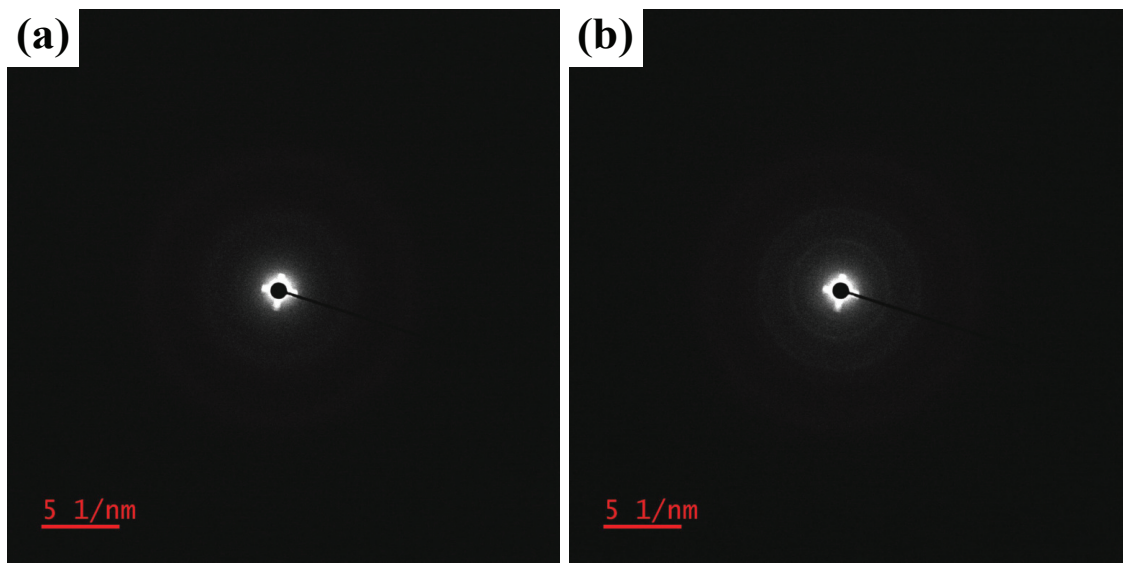

Figure S3 SAED pattern of (a) CD1 and (b) CD3.

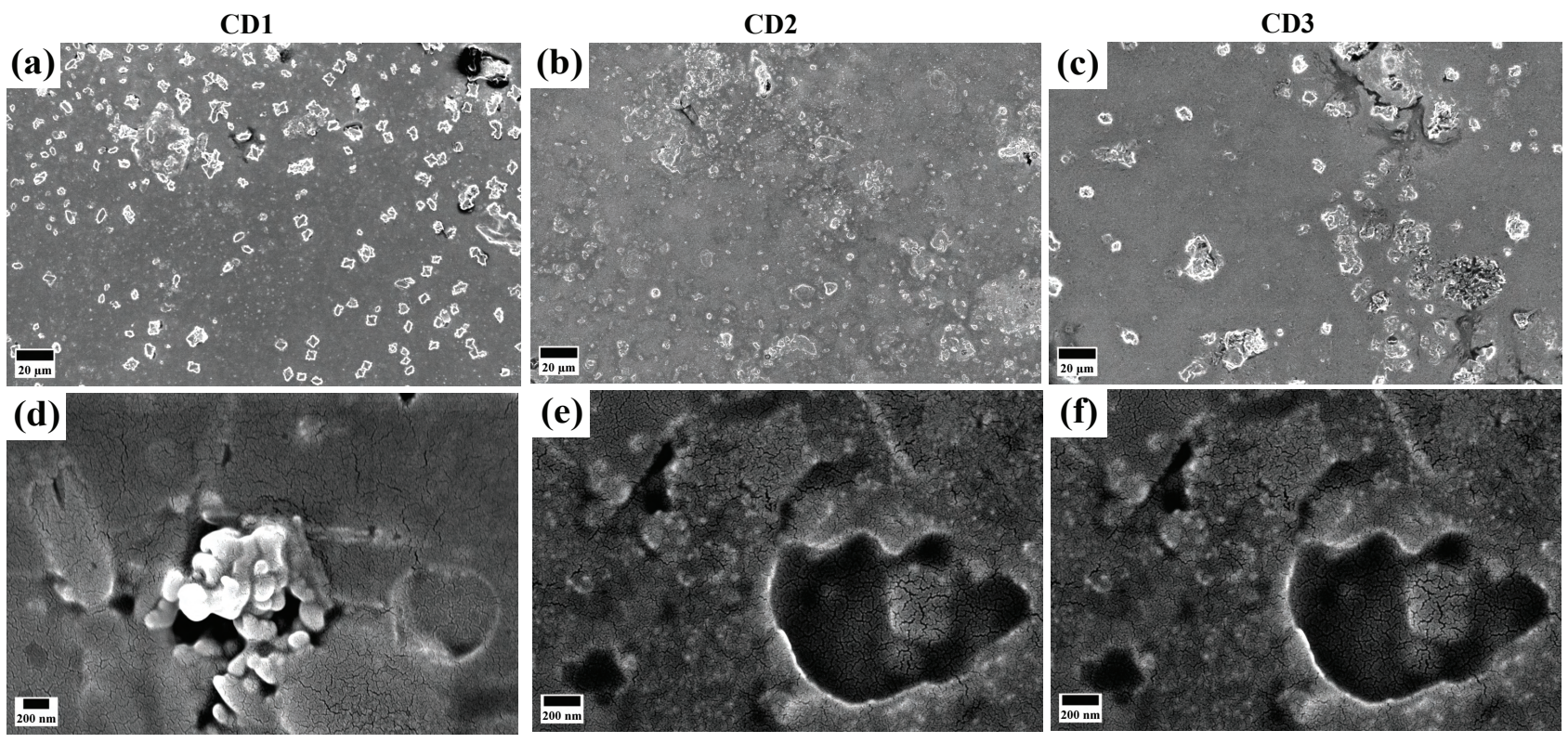

Figure S4 Low magnification SEM micrographs of (a) CD1, (b) CD2 and (c) CD3; high magnification FESEM micrographs of (d) CD1, (e) CD2 and (f) CD3.

3 EDAX analysis

Fig. S5(a), (b) and (c) represent the specific locations at which energy-dispersive X-ray spectroscopy (EDAX) was performed to record the corresponding elemental spectra of CD1, CD2, and CD3, as shown in Fig. S5(d), (e), and (f), respectively. The quantitative results presented in the insets confirm that CDs primarily consist of Carbon (C) and Oxygen (O), while the additional elemental peaks represent standard background signals from the measurement setup alongside native trace bio-elements originating from the mustard oil biomass precursor. Specifically, the dominant Silicon (Si) signal arises from the underlying silicon wafer substrate due to electron beam penetration through the thin drop-casted film, while the minor Aluminum (Al) peak could be originating from the metallic sample holder (Al stub) inside the chamber. Furthermore, the trace presence of elements such as Nitrogen (N), Calcium (Ca), Magnesium (Mg), Sodium (Na), etc. are attributed to the inherent phytochemicals, amino acids and essential mineral nutrients of the mustard seed biomass.[1]

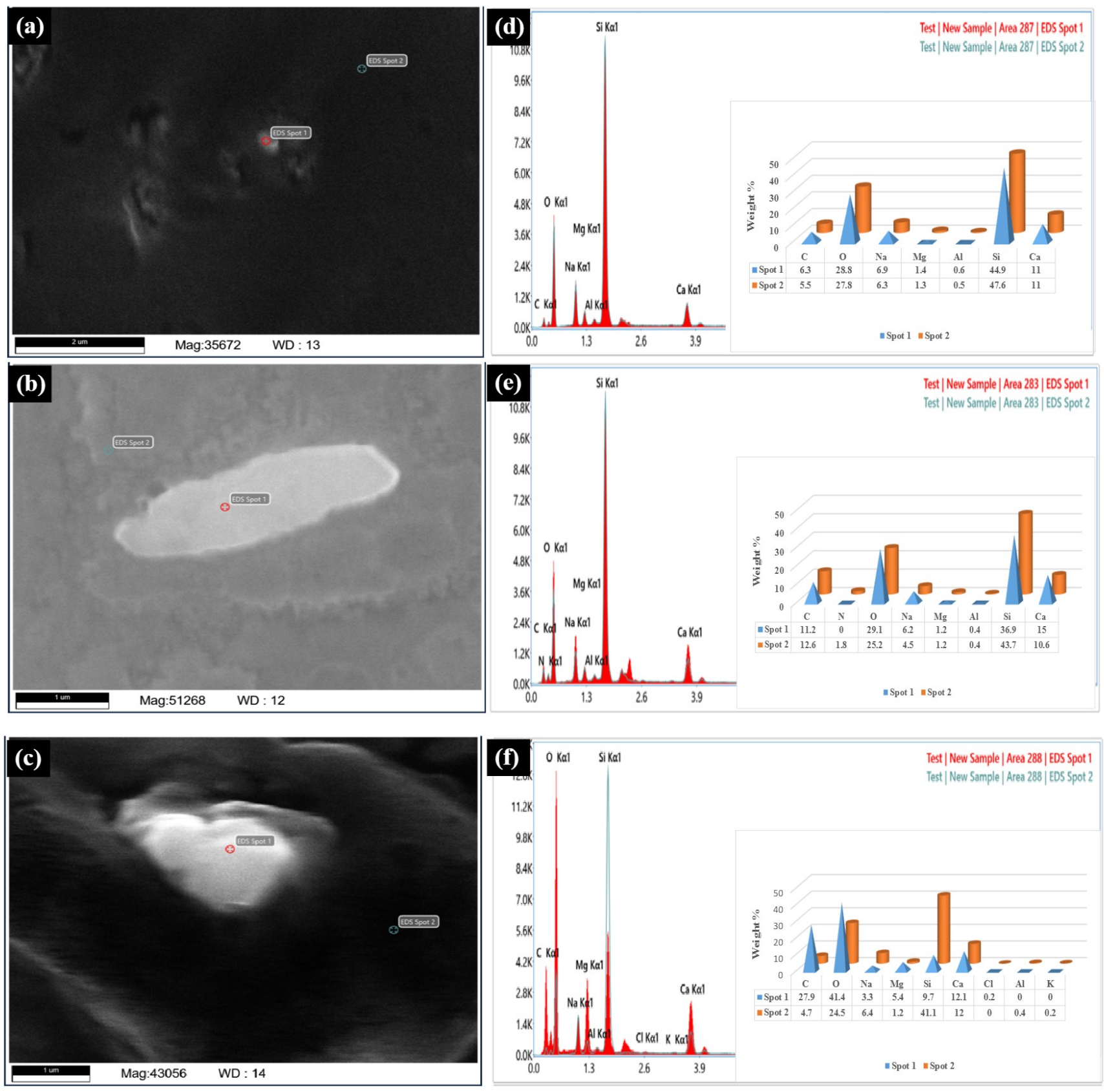

Figure S5 Site of EDAX measurement: (a)CD1, (b) CD2, (c) CD3; EDAX spectra of CS-CDs: (d) CD1, (e) CD2, (f) CD3 and their corresponding elemental graph in the inset.

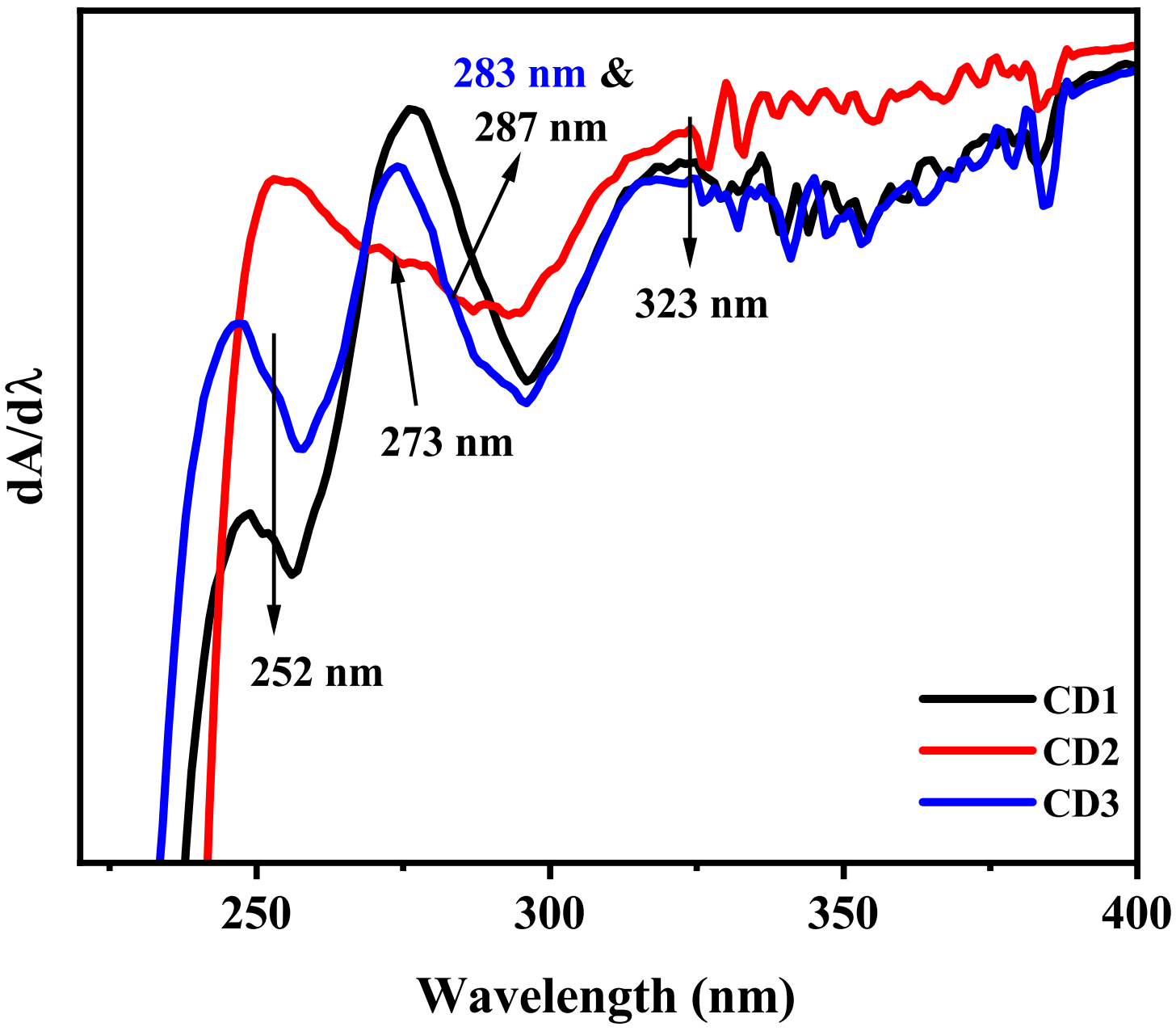

Figure S6 First derivative of UV-Vis absorption spectra of CS-CDs: CD1 (Black), CD2 (Red) and CD 3 (Blue).

4 Optical study: extended

The first-derivative absorption spectra ($dA/d\lambda$) of all three CS-CDs samples (CD1, CD2 and CD3) are shown in Fig.S6. The derivative spectra clearly resolve the underlying transition features. As evident from the UV-Vis absorption spectra in Fig. 4(a) and corroborated by the first-derivative analysis, characteristic transition regions consistently observed are: (i) a pronounced transition in the 250–290 nm region, attributed to π-π^* transition of aromatic sp^2 carbon domains and (ii) a broad shoulder peak centered at 323 nm region, corresponding to n–π^* transition associated with surface carbonyl (C=O) functional groups.

5 Reproducibility and stability assessment

The volume dependent emission shifts from 422 nm (1 L-CD1) to 430 nm (0.5 L-CD2) and 428 nm (2 L-CD3). We note that these shifts are highly reproducible and quantifiable in a batch of two (experiment performed in duplicates (n=2)), as can be seen from Fig.S7. Further, to ensure that the observed optical properties are independent of the specific brand of oil used as a precursor, control experiments were conducted using two commercial oil brands: oil 1 (Fortune) and oil 2 (Parmatma). Upon excitation at 350 nm, the normalized PL spectra of the resulting CDs from both oil sources exhibited identical peak positions (Fig.S8). These results demonstrate that the optical outcomes are highly reproducible and quantifiable irrespective of the oil source.

To evaluate the long-term stability of the soot-derived CS-CDs, the PL study was performed on CD3 which was stored for 8 months in refrigerator (Fig.S9). The PL spectrum was compared with that of day 1 sample and no appreciable change in PL peak position or intensity was observed. These results confirm that as-synthesized CDs retain their optical properties over time, manifesting their suitability for long-term storage and practical applications.

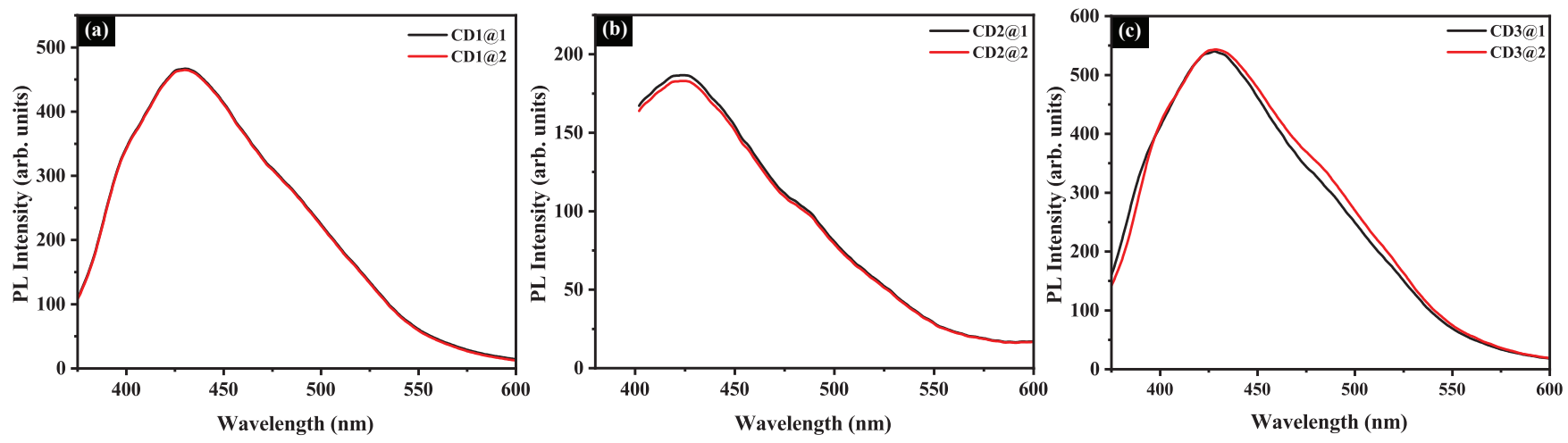

Figure S7 PL spectra of CS-CDs: (a) CD1, (b) CD2 and (c) CD 3 performed in duplicates (n=2).

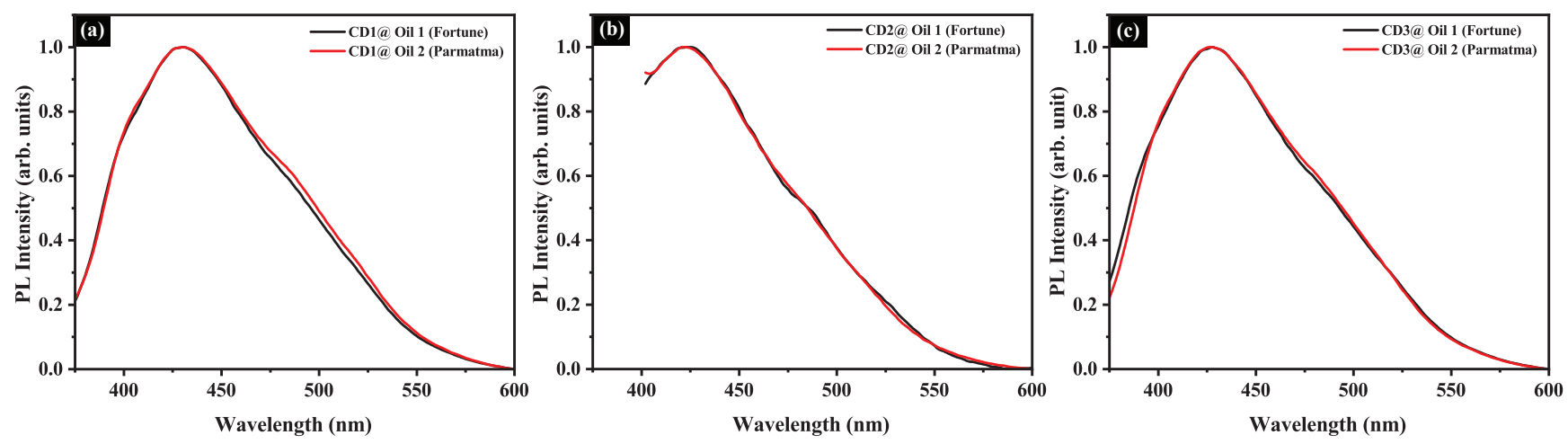

Figure S8 The comparison of normalized PL spectra of CS-CDs synthesized using two different commercial oil brands: oil 1 (Fortune) and oil 2 (Parmatma); (a) 0.5 L-CD1, (b) 1 L – CD2 and (c) 2 L – CD3. It shows the PL emission at (a) 430 nm (b) 422 nm and (c) 428 nm for CD1, CD2 and CD3, respectively upon excitation at 350 nm.

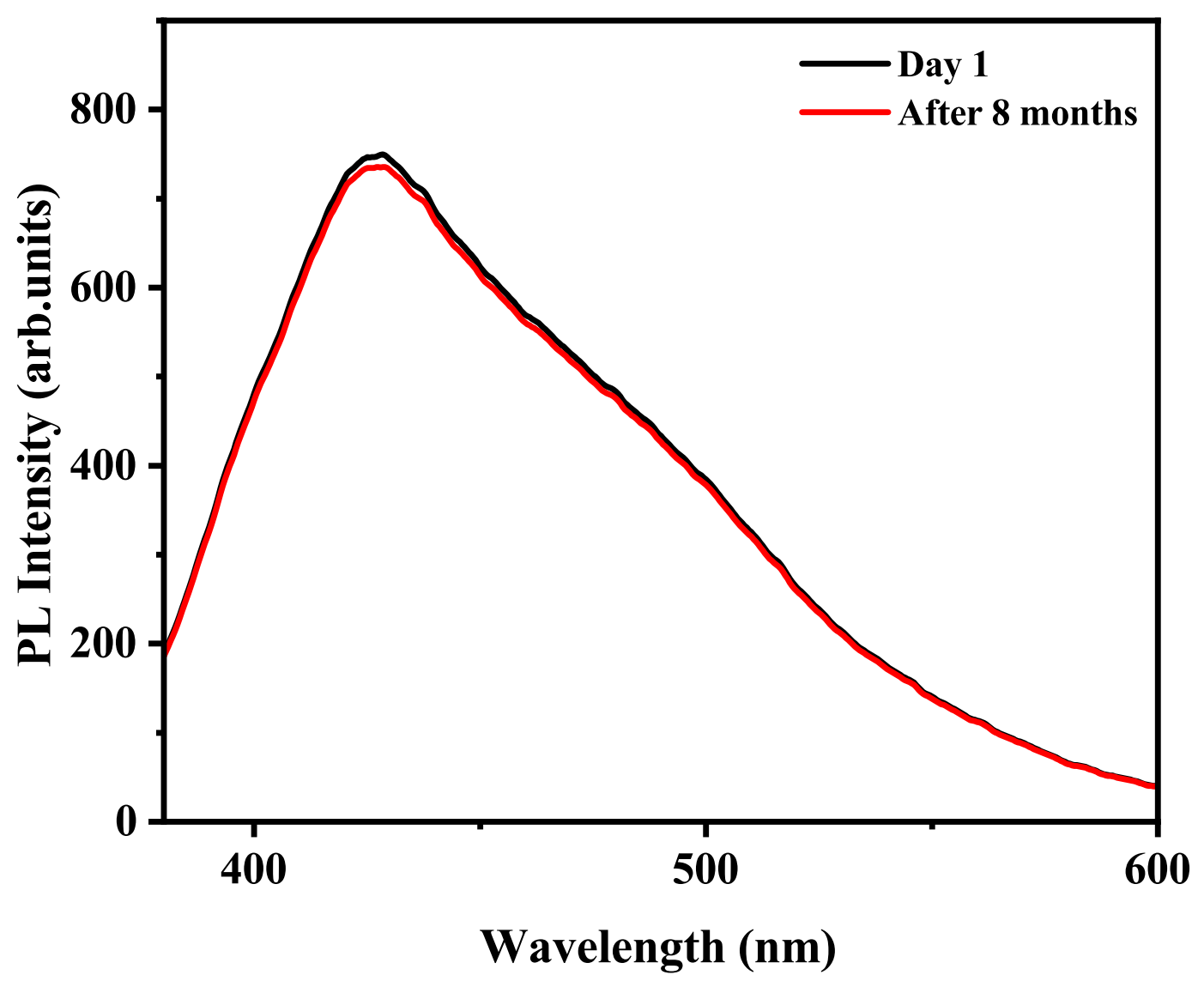

Figure S9 PL stability study of CD3 with black curve representing day 1 PL and red curve representing PL after 8 months of storage.

6 Selectivity of Pb^{2+} at 10 μM

The selectivity of blue-fluorescent CD3 was evaluated against various metal ions (Ca^{2+}, Bi^{2+}, Fe^{3+}, Mg^{2+}, Sr^{3+}, Pb^{2+},Cd^{2+}, As^{3+}, Al^{3+} and Hg^{2+}) at a higher concentration of 10 μM (Fig. S10). Although all ions exhibit varying degrees of PL quenching, Pb^{2+} demonstrates the highest quenching efficiency. However, at 10 μM, active surface site saturation occurs, leading to non-specific competitive interactions with other cations and a narrower relative quenching differential compared to 1 nM concentration regime.

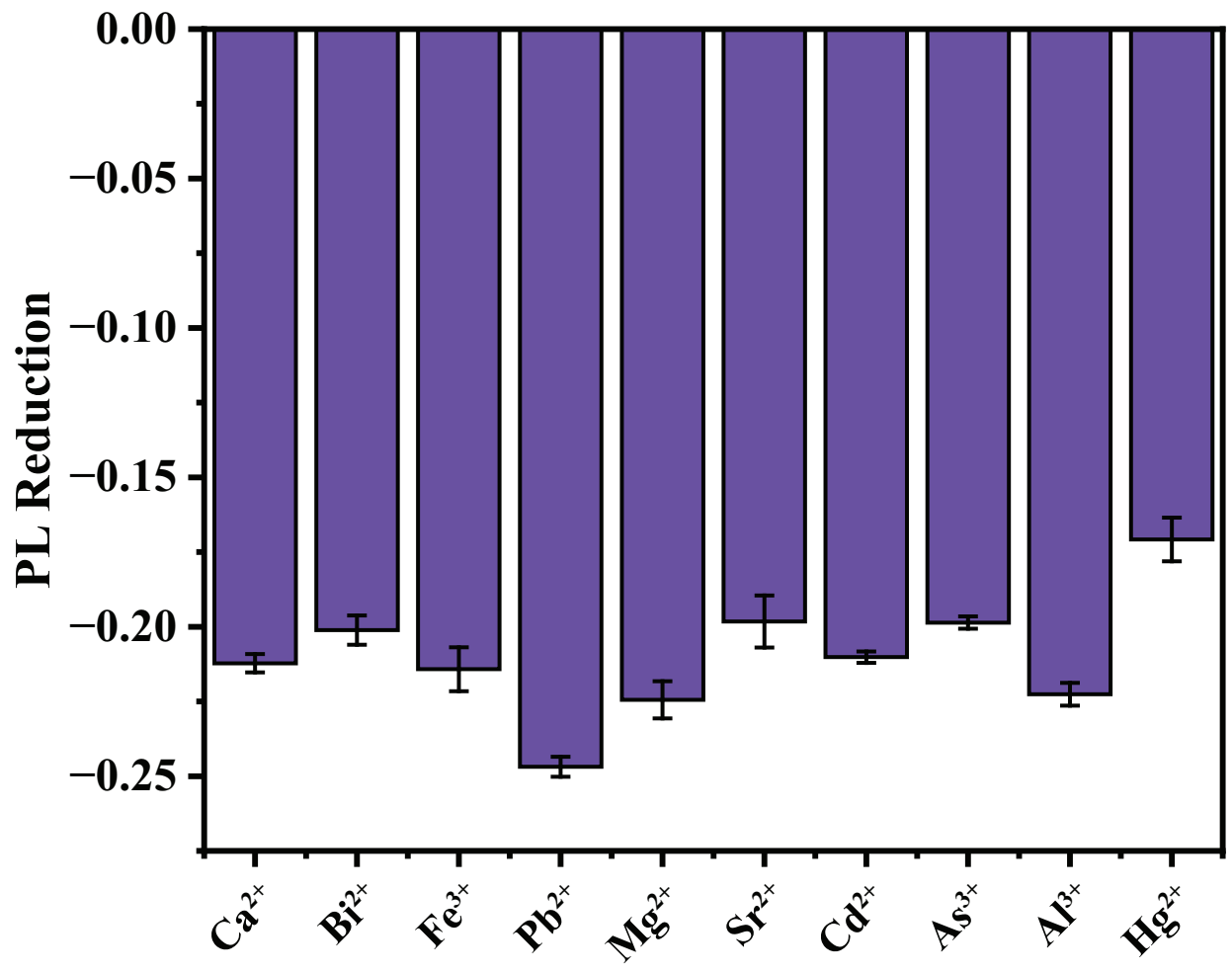

Figure S10 Histogram showing relative PL intensity of CDs when mixed with 10 μM concentration of different meta ions.

7 Phase contrast microscopy

Fig.S11(a) shows phase contrast microscopy of C2C12 cells when treated with lower concentration of CD1 and CD3. The analysis revealed that C2C12 cells maintained viability and normal morphology up to 250 μg/mL, exhibiting no discernible cytotoxic effects compared to untreated controls. Fig.S11(b) hows phase contrast microscopy of C2C12 cells after 48 hrs of CD1 and CD3 treatment. It clearly shows the overconfluency of the cells and its detachment from the surface.

Notes and references

[1] Y. Hu and Z. Yan, *Frontiers in Nutrition*, 2025, **12**, 1626333.

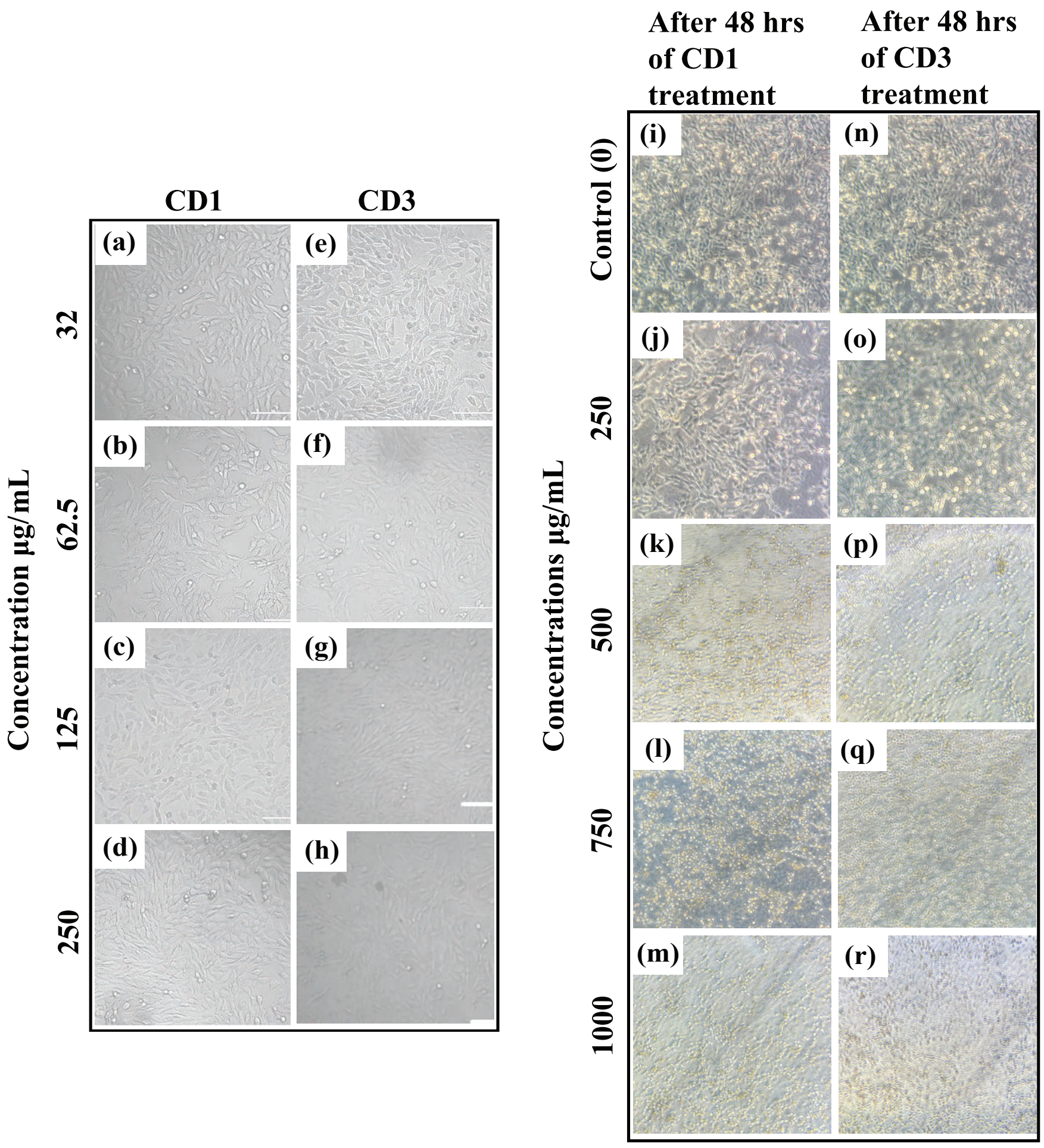

Figure S11 Phase contrast microscopy of C2C12 cells after treatment with CS-CDs 32, 62.5, 125 and 250 μg/mL: (a-d) cells incubated with CD1 and (e-h) cells treated with CD3. Phase contrast microscopy of C2C12 cells after 48 hrs treatment with CD1 and CD3-250, 500, 750 and 1000 μg/mL: (i and n) untreated cells 48 hrs after plating, (j-m) cells incubated for 48 hrs with CD1 and (o-r) cells incubated for 48 hrs with CD3, collectively showing enhanced cell growth at all concentrations.
